# The circular RNA circHMGB2 drives immunosuppression and anti-PD-1 resistance in lung adenocarcinomas and squamous cell carcinomas via the miR-181a-5p/CARM1 axis

**DOI:** 10.1186/s12943-022-01586-w

**Published:** 2022-05-07

**Authors:** Ling-Xian Zhang, Jian Gao, Xiang Long, Peng-Fei Zhang, Xin Yang, Shu-Qiang Zhu, Xu Pei, Bai-Quan Qiu, Shi-Wei Chen, Feng Lu, Kun Lin, Jian Jun Xu, Yong-Bing Wu

**Affiliations:** 1 Department of Cardiothoracic Surgery, the Second Affiliated Hospital of Nanchang University, 1 Ming de Road, Nanchang, 330000 Jiangxi People’s Republic of China; 2 Department of Thoracic Surgery, The Affiliated Zhongshan Hospital of Fudan University, Shanghai, 200032 People’s Republic of China; 3 Department of Medical Oncology, Zhongshan Hospital, Fudan University, Shanghai, 200032 People’s Republic of China

**Keywords:** circHMGB2, CARM1, NSCLC, TME, Anti-PD-1 treatment

## Abstract

**Background:**

Previous studies have confirmed the oncogenic role of HMGB2 in various cancers, but the biological functions of HMGB2-derived circRNAs remain unknown. Thus, we intended to investigate the potential role of HMGB2-derived circRNAs in lung adenocarcinomas (LUAD) and squamous cell carcinomas (LUSC).

**Methods:**

The expression profiles of HMGB2-derived circRNAs in LUAD and LUSC tissues and matched normal tissues were assessed using qRT–PCR. The role of circHMGB2 in the progression of the LUAD and LUSC was determined in vitro by Transwell, CCK-8, flow cytometry and immunohistochemistry assays, as well as in vivo in an immunocompetent mouse model and a humanized mouse model. In addition, in vivo circRNA precipitation assays, luciferase reporter assays and RNA pulldown assays were performed to explore the underlying mechanism by which circHMGB2 promotes anti-PD-1 resistance in the LUAD and LUSC.

**Results:**

The expression of circHMGB2 (hsa_circ_0071452) was significantly upregulated in NSCLC tissues, and survival analysis identified circHMGB2 as an independent indicator of poor prognosis in the LUAD and LUSC patients. We found that circHMGB2 exerted a mild effect on the proliferation of the LUAD and LUSC cells, but circHMGB2 substantially reshaped the tumor microenvironment by contributing to the exhaustion of antitumor immunity in an immunocompetent mouse model and a humanized mouse model. Mechanistically, circHMGB2 relieves the inhibition of downstream CARM1 by sponging miR-181a-5p, thus inactivating the type 1 interferon response in the LUAD and LUSC. Moreover, we found that the upregulation of circHMGB2 expression decreased the efficacy of anti-PD-1 therapy, and we revealed that the combination of the CARM1 inhibitor EZM2302 and an anti-PD-1 antibody exerted promising synergistic effects in a preclinical model.

**Conclusion:**

circHMGB2 overexpression promotes the LUAD and LUSC progression mainly by reshaping the tumor microenvironment and regulating anti-PD-1 resistance in the LUAD and LUSC patients. This study provides a new strategy for the LUAD and LUSC treatment.

**Supplementary Information:**

The online version contains supplementary material available at 10.1186/s12943-022-01586-w.

## Introduction

Lung cancer remains one of the most common malignancies, and it is the leading cause of cancer-related death in the world; non-small cell lung cancer (NSCLC) mainly including lung adenocarcinomas (LUAD) and squamous cell carcinomas (LUSC) accounts for approximately 85% of all cases of lung cancer [1]. While curative resection by lobectomy or segmentectomy has achieved promising effects in patients with early NSCLC, the overall prognosis of NSCLC remains poor; the overall 5-year survival rate is only 16% since most patients are diagnosed at an advanced stage due to the lack of symptoms of early NSCLC [1, 2]. Thus, further investigation of the oncogenesis and progression of NSCLC could have potential clinical value and may facilitate the development of new therapeutic strategies for patients with NSCLC.

Circular RNAs (circRNAs) are a class of regulatory RNAs that are characterized by a covalently closed loop structure; circRNAs are generated by the backsplicing of exons in precursor mRNA [3]. Previous studies have revealed the potential effects of circRNAs in various biological processes, such as cardiovascular disease [4], immunity [5] and cancer [6]. The biological functions and relevant mechanisms of circRNAs in various cancers have been thoroughly investigated, and the findings have provided new directions for the development of treatment strategies and cancer biomarkers. For instance, circRanGAP1 facilitates the progression of gastric cancer via the miR-887-3p/VEGFA axis [7]. Circ-cRAPGEF5 inhibits the invasion of renal cell carcinoma cells by sponging miR-27a-3p to block the inhibition of the downstream target TXNIP [8]. While some studies have reported a role of circRNAs in the oncogenesis and progression of NSCLC, the potential roles of most circRNAs in the development of NSCLC remain unclear and require further investigation [6].

High-mobility group box 2 (HMGB2) is a ubiquitous nuclear protein in humans that is responsible for the activation of chromatin domains [9]. HMGB2 is highly conserved and has universal biological characteristics, such as its ability to bind to DNA without sequence specificity [10]. Previous studies have identified HMGB2 as an oncogene that is associated with the poor prognosis of various cancers, including hepatocellular carcinoma, breast cancer and NSCLC [11–13]. Thus, we wondered whether the circRNAs transcribed by HMGB2 contribute to the progression of NSCLC (here indicated LUAD and LUSC). In this study, we compared the expression profiles of circRNAs transcribed by HMGB2 in NSCLC tissues and paired normal tissues and observed a significant upregulation of circHMGB2 (hsa_circ_0071452) in NSCLC tissues. Interestingly, the high expression of circHMGB2 indicated a poor prognosis of NSCLC patients and contributed to the malignant properties of NSCLC by supporting an immunosuppressive microenvironment. Mechanistically, HMGB2 sponged miR-181a-5p and further blocked the inhibition of the downstream molecule CARM1, which is responsible for the inactivation of the type 1 interferon (IFN) response; thus, HMGB2 led to the immune evasion of NSCLC. Therefore, this study presents circHMGB2 as a potential molecular target for immunotherapy in NSCLC.

## Methods

### Cell lines

The cell lines used in this study included the human NSCLC cell lines A549, PC-9, NCI-H460, NCI-H1299, NCI-H1703 and human bronchial epithelial (HBE) cells. The mouse lung cancer cell lines LLC and HEK-293 T were obtained from the Cell Bank of the Chinese Academy of Sciences (Shanghai, China). The cells were cultivated in DMEM and RPMI-1640 (Gibco, USA) supplemented with 10% fetal bovine serum (Gibco, USA) and 1% penicillin–streptomycin (Yeasen, Shanghai, China). The environmental conditions were 37 °C and 5% CO_2_.

### Patients and follow-up

A total of 120 pairs of NSCLC tissues that were identified by two experienced pathologists were collected from patients who underwent lobectomy or segmentectomy at the Second Affiliated Hospital of Nanchang University from 2013 to 2014. These tissues were used to construct a tissue microarray (TMA). The TMA was used to perform immunohistochemistry (IHC) staining. The last follow-up occurred in June 2019. The Ethics Committee of the Second Affiliated Hospital of Nanchang University approved the human ethics-related protocols of this study, and informed consent was obtained from all the patients.

### Agarose gel electrophoresis, Western blotting, quantitative real-time polymerase chain reaction (qRT–PCR), IHC, fluorescence in situ hybridization (FISH), wound healing assays, Matrigel Transwell assays, cell counting Kit-8 (CCK-8) assays and colony formation assays

These experiments were performed following protocols described in previous studies [14], and the details are provided in the Supplementary Methods and Materials. Information about the antibodies and primers is presented in supplementary Tables 1 and 2. The probe sequences for circHMGB2 and miR-181a-5p are listed in Supplementary Table 3.

### Transfection of lentiviral vectors and generation of CRISPR Cas9-edited LLC and A549 cells

The lentiviral vectors carrying circHMGB2 and shcircHMGB2 were purchased from Genomeditech (Shanghai, China) and used to establish stably transfected cell lines. The transfection efficiency was assessed by qRT–PCR. Knockout of the CARM1 gene in the LLC and A549 cell lines via the CRISPR Cas9-gDNA system was accomplished by Genomeditech, and western blotting was used to determine the efficacy. The sequences of shcircHMGB2 and sgCARM1 are listed in Supplementary Tables 4 and 5.

### In vivo circRNA precipitation (circRIP) and RNA immunoprecipitation (RIP)

The experimental procedures for the circRIP and RIP assays were described in a previous study [14]. For the circRIP assay, the biotin-labeled circHMGB2 probe was purchased from Gene-Chem (Shanghai, China). Briefly, after transfection with the biotin-circHMGB2 and NC probes, H1299 cells were fixed with 4% formaldehyde, lysed, sonicated and centrifuged. Next, the supernatant was incubated with M280 streptavidin Dynabeads (Invitrogen) for 12 h. Then, the mixture was washed and suspended in lysis buffer. The total RNA was extracted from the mixture with TRIzol reagent (Invitrogen).

For the RIP assay, the Magna RIP kit (Millipore, USA) was used to enrich circHMGB2 and miRNA. Total RNA was extracted with TRIzol reagent, and the levels of the target circRNAs and miRNAs were measured by qRT–PCR.

### RNA pull-down assay and co-IP combined with MS

The pull-down assay was performed according to a previous study [15]. First, the biotinylated miR-181a-5p mimics and negative control (NC) mimics were mixed with M-280 streptavidin magnetic beads (Invitrogen) and incubated for approximately 2 h. Then, H1299 cells were lysed and incubated with the mixture for 12 h. Finally, the RNAs sponged by the beads were extracted and analyzed by qRT–PCR.

The immunoprecipitation was performed in H1299, A549 and PC9 cells, and the CARM1 antibody preabsorbed protein A- and G-Sepharose beads was used as the primary antibody. The 2D-LC-MS/MS protocol was performed as previously described [16].

### Dual-luciferase reporter gene assay

pGL3-LUC-circHMBG2, pGL3-LUC-CARM1, mutant pGL3-LUC-circHMGB2, and mutant-pGL3-LUC-CARM1 were co-transfected with miR-181a-5p mimics or NC mimics into HEK-293 T cells for 48 h. Then, the cells were lysed and centrifuged, and the supernatants were collected. The luciferase activities in the supernatants were measured using a dual-luciferase reporter assay system (Promega). The activation of the target gene was calculated according to the ratio of firefly luciferase activity/Renilla luciferase activity.

### Flow cytometry analysis

The immune cell profiles in subcutaneous tumors were investigated by flow cytometry. Fresh tumor tissues were homogenized into single cell suspensions, and immune cells were isolated with Percoll. For surface marker staining, 1 × 10^6^ cells were washed with 2 ml staining buffer (PBS with 1% FBS and 0.2% EDTA) and centrifuged at 350 g × 6 min. Then, the cells were incubated with Fc blocker and antibodies for 30 min. For the staining of Foxp3 in the nucleus, after surface marker staining for 30 min, the cells were washed with 1 ml staining buffer, fixed with 4% paraformaldehyde for 15 min and permeabilized with 0.1% Triton X-100 for 30 min. Next, the cells were washed with 1 ml staining buffer and incubated with Fc blocker and anti-Foxp3 antibodies for 90 min. Flow cytometry analysis was performed with a Fortessa flow cytometer (BD Biosciences, USA). Information about the antibodies is listed in Supplementary Table 1, and the CD45^+^CD3^+^CD8^+^ indicates CD8 cells; CD45^+^CD3^+^CD4^+^, CD4 cells; CD45^+^CD3^−^NK1.1/CD56^+^, NK; CD45^+^CD3^+^CD4^+^FOXP3^+^, Treg; CD45^+^CD11b^+^F4/80^+^ and DC, CD45^+^CD11b^−^CD11c^+^ TAM.

### Exosome extraction and electron microscopy

Exosomes in the supernatants of NSCLC cells were extracted by ExoQuick Exosome Precipitation Solution (SBI System Biosciences) according to the manufacturer’s protocol. The exosomes were further identified by electron microscopy and western blotting.

### Humanized mouse generation

The huHSC-NOG-EXL mice were purchased from Beijing Vital River Laboratory. New-born NOG-EXL mice were irradiated with 2Gy and subsequently injected with 5 × 10^4^ CD34^+^ human hematopoietic stem cells (HSCs) through the tail vein. After 7–8 weeks of HSC differentiation, the reconstitution of human immune system components in the peripheral blood of humanized NOG-EXL mice was analyzed. A proportion of hCD45^+^ cells higher than 45% was considered to indicate the successful establishment of huHSC-NOG-EXL mice. Then, tumor engraftment was performed in weeks 8–11 (Fig. 4E).

### In vivo tumor growth and anti-PD-1 therapy

C57BL/6 mice were purchased from Jiesijie (Shanghai, China). The C57BL/6 mice and huHSC-NOG-EXL mice were fed in a pathogen-free environment in the Center for Experimental Animals of Zhongshan Hospital. The animal experiments were approved by the Ethics Committee of Zhongshan Hospital. Approximately 5 × 10^6^ cells were resuspended in 150 μl DMEM medium and subcutaneously injected into the right flanks of the C57BL/6 mice or huHSC-NOG-EXL mice. When the tumor size reached 100mm^3^, the PD-1 antibody or IgG was administered i.p. at a dose of 100 μg/injection every three days. The CARM1 inhibitor EZM2302 was administered orally at a dose of 150 mg/kg twice a day for 14 days [17]. The tumor size was assessed every 3 days.

### Statistical analysis

Data analysis was performed with 23.0 SPSS (Chicago, IL). The values are shown as the mean ± standard deviation. Student’s t test was chosen to compare differences in measurement data between two groups, while categorical variables were compared via chi-squared or Fisher’s exact tests. Spearman correlation analysis was used to analyze correlations between the levels of circHMGB2, miR-181a-5p, CARM1, CD8, CD56, NK1.1 (CD161), CD11C, p-STAT1, ISG15 and IFIT1. The Kaplan–Meier method and the log-rank test were used to analyze differences in the prognosis of NSCLC patients. Cox’s regression model was used to investigate independent prognostic factors. All the *p* values were two tailed, and differences with *p* < 0.05 were considered statistically significant.

## Results

### Clinical significance of circHMGB2 in NSCLC patients

**Fig. 1:**
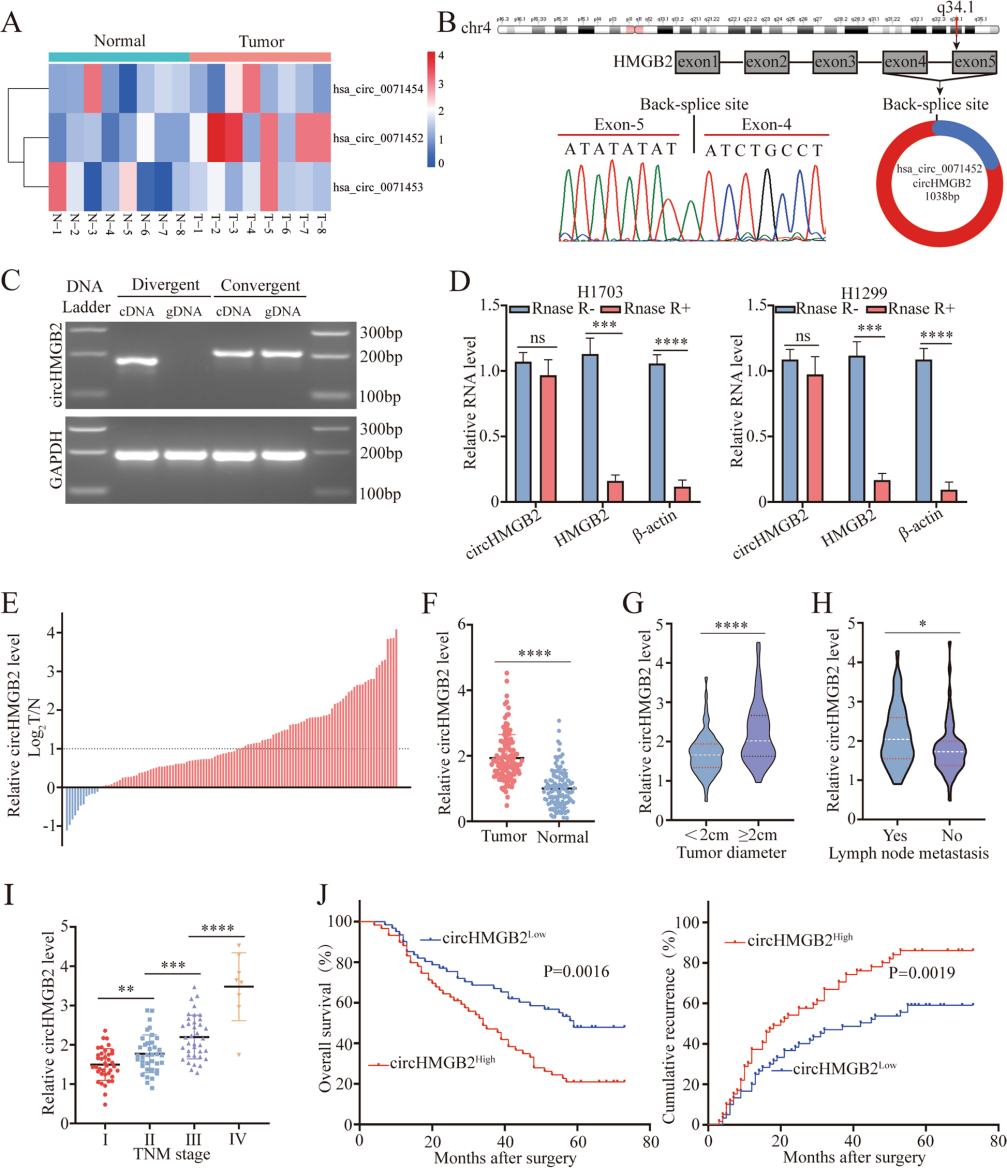
The clinical significance of circHMGB2 in NSCLC patients. **A** The expression of HMGB2-derived circRNAs was measured in 8 pairs of NSCLC tissues and matched normal tissues. **B** Schematic diagram of circHMGB2 and Sanger sequencing results of the site of circHMGB2 backsplicing. **C** PCR results of circHMBG2 transcribed with divergent or convergent primers from cDNA and gDNA. **D** circHMGB2 and HMGB2 in H1703 and H1299 cell lines were detected by qRT–PCR after digestion by RNase. **E** and **F** The expression of circHMGB2 was detected by qRT–PCR in 120 pairs of NSCLC tissues and matched normal tissues. **G** and **H** The expression of circHMGB2 was analyzed according to tumor diameter (< 2 cm vs. ≥ 2 cm) and lymph node metastasis status (yes vs. no). **I** The expression of circHMGB2 was analyzed according to TNM stage. **J** Survival analysis of the recurrence and OS of 120 NSCLC patients divided into groups according to circHMGB2 expression (circHMGB2^high^ vs. circHMBG2^low^) was performed using Kaplan–Meier and log rank analysis. Data are presented as the means ± SD of three independent experiments. *P < 0.05, **P < 0.01, *** *P* < 0.001, ***P < 0.0001, ns: not significant

HMGB2 is reported to be involved in the progression of many types of cancers, including NSCLC [11–13, 18]. In general, the expression of circRNAs is closely related to the expression of the corresponding mRNAs since circRNAs are derived from the back-splicing of precursor mRNAs [3]. Thus, the expression of three circRNAs derived from HMGB2 was measured in 8 pairs of NSCLC tissues and paired normal tissues via qRT–PCR. The results showed that hsa_circ_0071452 (circHMGB2) was the most significantly upregulated circRNA in NSCLC tissues (Fig. 1A and Supplementary Fig. 1A). Sanger sequencing with divergent primers also identified the loop structure of circHMGB2 (Fig. 1B). In addition, PCR results showed that circHMGB2 could be transcribed with divergent primers only from cDNA but not from gDNA (Fig. 1C). After incubation with RNase R for 30 min, the qRT–PCR results showed that circHMGB2 was resistant to RNase R, while the mRNA of HMGB2 and β-actin as positive control was substantially degraded (Fig. 1D).

To further evaluate the clinical role of circHMGB2 in NSCLC, the level of circHMGB2 was measured in 120 NSCLC tissues and matched normal tissues using qRT–PCR. CircHMGB2 was highly expressed in the majority of tumor tissues, and the expression of circHMGB2 in 57 NSCLC tissues was more than twofold higher than that in normal tissues (Fig. 1E and F). The relationships between the level of circHMGB2 and the clinical characteristics of 120 NSCLC patients are presented in Table 1. These results suggested that high expression of circHMGB2 was related to large tumor size and lymph node metastasis in NSCLC patients (Fig. 1G and H). We also observed that circHMGB2 expression was increased as clinical stage increased (Fig. 1I). Moreover, Kaplan–Meier analysis revealed that patients with high circHMGB2 expression had lower overall survival (OS) and higher postoperative recurrence rates (Fig. 1J) than those with lower circHMGB2 expression. Furthermore, multivariate Cox analysis confirmed that circHMGB2 was an independent factor in the prognosis of NSCLC patients (Tables 2 and 3). Additionally, we further analyzed the clinical implication of circHMGB2 in LUAD (Supplementary Fig. 1B and C). In short, these results suggest that high expression of circHMGB2 indicates a poor prognosis in NSCLC patients and is involved in the progression of NSCLC.

**Table 1:** Correlations between circRNA HMGB2 and clinical characteristics in 120 NSCLC patients

Clinicopathological parameters	No. of cases	CircHMGB2 expression level		*P* value
		Low	High	
Age				
≥60	53	28	25	
<60	67	32	35	0.581
Gender				
Male	67	36	31	
Female	53	24	29	0.358
Smoking history				
Smokers	54	24	30	
Nonsmokers	66	36	30	0.271
Histological type				
Adenocarcinoma	78	41	37	
Squamous	42	19	23	0.444
Tumor stage				
I–II	72	31	41	
III–IV	48	29	19	0.062
Lymph node metastasis				
Yes	50	19	31	
NO	70	41	29	0.026
Tumor size				
>2 cm	55	21	34	
≤2 cm	65	39	26	0.017
Differentiation				
Well and moderate	57	23	34	
Poor	63	37	26	0.044

**Table 2:** Univariate and multivariate analyses of factors associated with overall survival

Factors	OS					
	Univariate			Multivariate		
	HR	95% CI	*P* value	HR	95% CI	*P* value
Age(≥60 VS <60)	0.856	0.683–1.073	0.178			NA
Gender(Male VS Female)	0.914	0.726–1.151	0.446			NA
Smoking history (smokers VS Nonsmokers)	0.946	0.755–1.186	0.631			NA
Histological type (adenocarcinoma VS squamous)	1.082	0.676–1.733	0.741			NA
Tumor stage (III–IV VS I–II)	0.79	0.630–0.991	0.041	0.692	0.544–0.882	0.003
Lymph node metastasis (Yes VS No)	0.716	0.571–0.898	0.004	0.735	0.581–0.929	0.01
Tumor size (>2 cm VS ≤2 cm)	0.732	0.582–0.919	0.007	0.811	0.639–1.030	0.086
Differentiation (poor VS well and moderate)	0.75	0.597–0.943	0.014	0.664	0.517–0.851	< 0.001
CircHMGB2 expression (High vs. Low)	0.709	0.562–0.894	0.004	0.600	0.459–0.783	<0.001

**Table 3:** Univariate and multivariate analyses of factors associated with cumulative recurrence

Factors	OS					
	Univariate			Multivariate		
	HR	95% CI	*P* value	HR	95% CI	*P* value
Age(≥60 VS <60)	0.957	0.773–1.185	0.687			NA
Gender(Male VS Female)	0.931	0.750–1.156	0.518			NA
Smoking history (smokers VS Nonsmokers)	0.919	0.743–1.138	0.44			NA
Histological type (adenocarcinoma VS squamous)	0.985	0.629–1.544	0.949			NA
Tumor stage (III–IV VS I–II)	0.77	0.622–0.955	0.017	0.68	0.540–0.856	0.001
Lymph node metastasis (Yes VS No)	0.722	0.582–0.895	0.003	0.749	0.598–0.939	0.012
Tumor size (>2 cm VS ≤2 cm)	0.778	0.627–0.965	0.022	0.854	0.681–1.070	0.17
Differentiation (poor VS well and moderate)	0.782	0.630–0.970	0.025	0.711	0.564–0.896	0.004
CircHMGB2 expression (High vs. Low)	0.713	0.573–0.888	0.002	0.617	0.479–0.795	<0.001

### CircHMGB2 promotes the proliferation of NSCLC and reshapes the tumor microenvironment (TME)

**Fig. 2:**
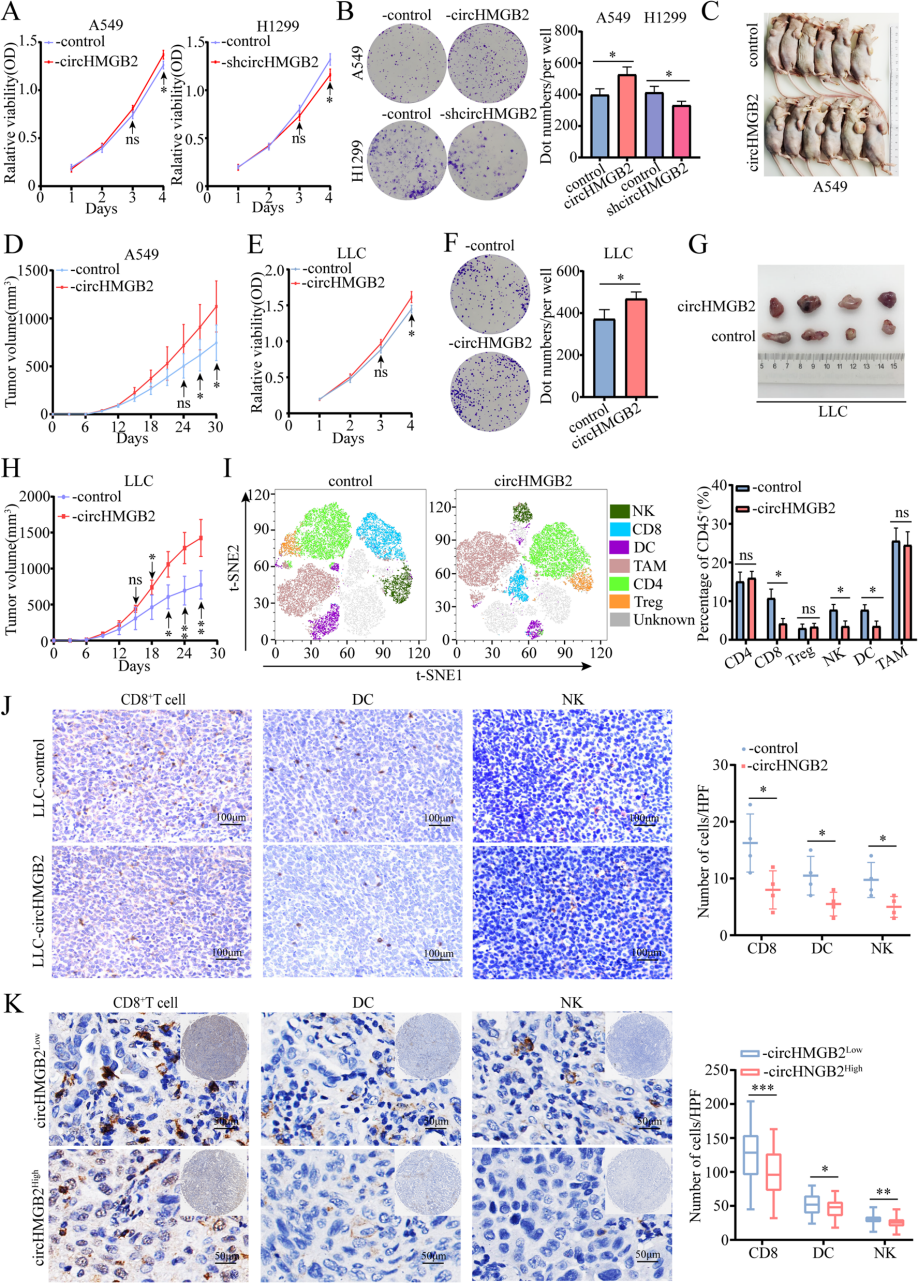
CircHMGB2 promotes the proliferation of NSCLC and reshapes the tumor TME. **A** The proliferation of A549-circHMGB2 and H1299-shcircHMGB2 cells was measured by CCK-8 assay. **B** The viability of A549-circHMGB2 and H1299-shcircHMGB2 cells was measured by colony formation assays. **C** and **D** Nude mice were subcutaneously implanted with A549-control and A549-circHMGB2 cells, and the tumor size was assessed every 3 days. **E** The proliferation of LLC-circHMGB2 cells was measured by CCK-8 assay. **F** The viability of LLC-circHMGB2 cells was measured by colony formation assays. **G** and **H** C57BL/6 mice were subcutaneously implanted with LLC-control and LLC-circHMGB2 cells, and the tumor size was assessed every 3 days. **I** The immune cell profiles of subcutaneous tumors derived from LLC-control and LLC-circHMGB2 cells were assessed by flow cytometry. **J** IHC staining of CD8^+^ T cells, DCs, and NK cells in subcutaneous tumors derived from LLC-control and LLC-circHMGB2 cells. **K** The infiltration of CD8^+^ T cells, DCs and NK cells in 120 NSCLC tissues divided into circHMGB2^low^ and circHMGB2^high^ groups was assessed by IHC. Data are presented as the means ± SD; *n* = 3, *P < 0.05, **P < 0.01, ***P < 0.001, ns: not significant

Considering the potential effect of circHMGB2 on the prognosis of NSCLC patients, the impact of circHMGB2 on the biological functions of NSCLC was further investigated. The expression of circHMGB2 in 5 NSCLC cell lines and LLC cells was measured by qRT–PCR, and the results showed that circHMGB2 was expressed at the highest level in H1299 cells and the lowest level in A549 cells (here only including LUAD and LUSC cell lines) (Supplementary Fig. 2A). Then, circHMGB2 expression was knocked down in H1299 cells (H1299-shcircHMGB2) and overexpressed in A549 and LLC cells (A549-circHMGB2, LLC-circHMGB2) by lentivirus transfection, and three stable cell lines were generated (Supplementary Fig. 2B). A matrigel transwell assay revealed no significant effect of circHMGB2 on the invasion of NSCLC cells (Supplementary Fig. 2C). Consistently, a wound healing assay showed no effect of circHMGB2 in inhibiting or promoting the migration of A549-circHMGB2 or H1299-shHMGB2 cells (Supplementary Fig. 2D). However, CCK-8 and colony formation assays showed that proliferation was decreased after the knocked-down of circHMGB2 and increased after the elevated circHMGB2 expression in NSCLC cells (Fig. 2A and B). In addition, the in vitro analysis of nude mice subcutaneously implanted with A549-circHMGB2 and control cells further showed that the overexpression of circHMGB2 could accelerate the growth of NSCLC (Fig. 2C and D).

Additionally, further studies were performed to determine whether circHMGB2 could potentially impact the TME. Firstly, CCK-8 and colony formation assays showed that proliferation was increased after circHMGB2 expression was upregulated in LLC cells (Fig. 2E and F). Then, we performed an in vivo assay in C57BL/6 mice via subcutaneously inject with LLC-circHMGB2 and LLC-control cells, and found that circHMGB2 could dramatically potentiate the growth of subcutaneous tumors in immunocompetent mice (Fig. 2G and H). Moreover, the immune profiles in the tumors were evaluated using flow cytometry. The results revealed the presence of exhausted of CD8^+^ T cells, NK cells and DCs in the subcutaneous tumors derived from LLC-circHMGB2 cells (Fig. 2I). The IHC staining of CD8^+^ T cells, CD4^+^T cells, NK1.1^+^ NK cells, CD68^+^TAM, FOXP3^+^Treg cells and CD11C^+^ DCs in the tumor tissues also showed a similar trend (Fig. 2J and Supplementary Fig. 2E). To further verify the in vivo results, IHC staining of CD8, CD56 and CD11C expression in 120 NSCLC tissues was performed, and the results revealed similar trends. The infiltration of CD8^+^ T cells, CD56^+^ NK cells and CD11C^+^ DCs was remarkably lower in NSCLC samples with high circHMGB2 expression (Fig. 2K). Considering the evidently effects of circHMGB2 on the NSCLC TME, we hypothesized that circHMGB2 induces the progression of NSCLC mainly by limiting antitumor immunity in the TME.

### CircHMGB2 upregulated the expression of the downstream molecule CARM1 by sponging miR-181a-5p

Further investigation of the relationship between circHMGB2 and the TME led to two possible hypotheses: (1) circHMGB2 is directly transmitted into the TME and affects the biological functions of immune cells or (2) the upregulation of circHMGB2 expression inhibits the immune response to NSCLC. Since exosomes have been reported to be a critical mechanism by which circRNAs are transmitted between cells [15, 19, 20], exosomes were extracted from the supernatants of H460-circHMGB2 cells to test the first hypothesis (Supplementary Fig. 3A and B). However, no markedly enrichment in the exosomes derived from H460-circHMGB2 cells was observed (Supplementary Fig. 3C). Therefore, further studies focused on the function of circHMGB2 in NSCLC.

**Fig. 3:**
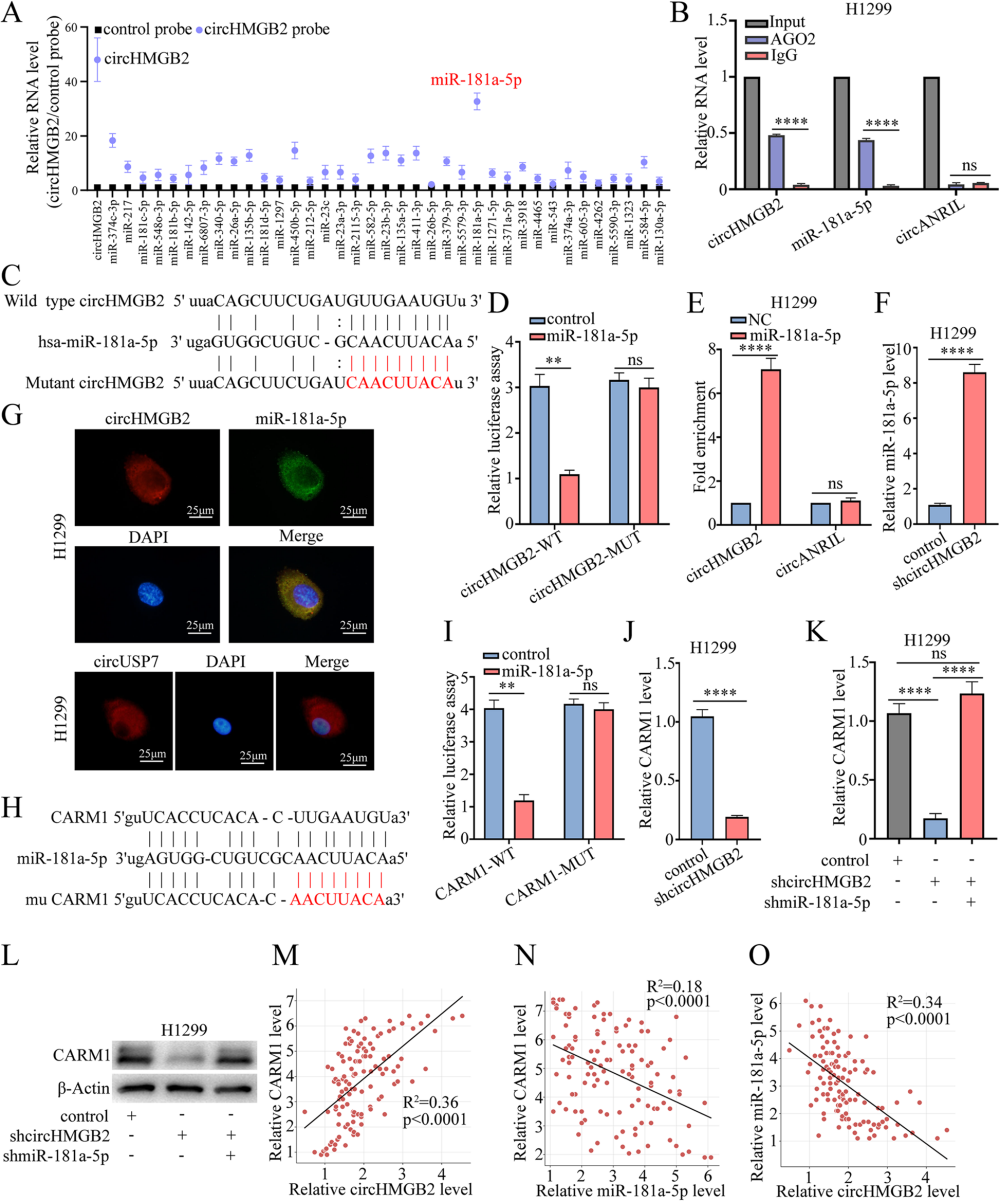
CircHMGB2 upregulates the expression of the downstream molecule CARM1 by sponging miR-181a-5p. **A** A circRIP assay was performed with a circHMGB2 probe in H1299 cells. **B** The RIP assay was performed using the anti-AGO2 antibody in H1299 cells. **C** The putative binding sites of circHMGB2 and miR-181a-5p. **D** The luciferase activity of circHMGB2 in HEK-293 T cells transfected with miR-181a-5p. **E** The RNA pulldown assay was performed with H1299 cells transfected with biotinylated miR-181a-5p. **F** The expression of miR-181a-5p was measured via qRT–PCR after the knockdown of circHMGB2 in H1299 cells. **G** FISH staining of circHMGB2 and miR-181a-5p in H1299 cells. circUSP7 as positive control **H** The putative binding sites of CARM1 and miR-181a-5p. **I** The luciferase activity of CARM1 was measured in HEK-293 T cells transfected with miR-181a-5p. **J** The expression of CARM1 was measured via qRT–PCR after the overexpression of circHMGB2. **K** The expression of CARM1 was measured using qRT–PCR after the dual knockdown of circHMGB2 and miR-181a-5p expression. **L** The expression of CARM1 was measured using western blotting after the dual knockdown of circHMGB2 and miR-181a-5p expression. **M**-**O** The relationships among the expression levels of circHMGB2, miR-181a-5p and CARM1 in 120 NSCLC tissues were assessed using qRT–PCR. Data are presented as the means ± SD; *n* = 3, **P < 0.01, ****P < 0.001, ns: not significant

Since circRNAs have been widely reported to act as competing endogenous RNAs (ceRNAs) for miRNAs, potential miRNAs that could be sponged by circHMGB2 were predicted using StarBase 3.0. Then, circRIP with a circHMGB2 probe was performed in H1299 cells, and the significant enrichment of miR-181a-5p was observed via qRT–PCR (Fig. 3A). Further verification of the interaction between circHMGB2 and miR-181a-5p using RIP revealed the remarkable enrichment of circHMGB2 and miR-181a-5p by an anti-AGO_2_ antibody (Fig. 3B). Moreover, miR-181a-5p mimics were co-transfected with a luciferase plasmid carrying the wild-type circHMGB2 sequence and a mutant sequence into HEK-293 T cells. The miR-181a-5p mimics obviously weakened the luciferase activity of the wild-type circHMGB2 sequence but not the luciferase activity of the mutant circHMGB2 sequence (Fig. 3C and D). The miR-181a-5p pulldown assay also revealed significant enrichment of circHMGB2 (Fig. 3E). In addition, the qRT–PCR results showed that the level of miR-181a-5p was elevated after the knockdown of circHMGB2 expression in H1299 cells (Fig. 3F). FISH staining of circHMGB2 and miR-181a-5p in H1299 cells showed that these molecules colocalized in the cytoplasm (Fig. 3G). Thus, circHMGB2 may perform its biological function by sponging miR-181a-5p. To identify the downstream mRNAs, StarBase 3.0, miRanda and PITA were used to predict the potential targets of miR-181a-5p, and the results showed that the immune-related genes SIRT1 [21], CDK8 [22], IRS2 [23], BHLHE40 [24] and CARM1 [25] contained potential binding sites for miR-181a-5p. Furthermore, the CARM1 mRNA was enriched by biotin-miR-181a-5p in NSCLC cells (Supplementary Fig. 4A). Subsequently, a luciferase reporter assay showed that the luciferase activity of the wild-type CARM1 sequence was reduced compared to that of the mutant CARM1 sequence (Fig. 3H, I and Supplementary Fig. 4B-E). qRT–PCR also showed that the expression of CARM1 was reduced in H1299-shcircHMGB2 cells (Fig. 3J), while the knockdown of miR-181a-5p expression in H1299-shcircHMGB2 cells significantly restored the expression of CARM1 (Fig. 3K and L). Moreover, the levels of circHMGB2, miR-181a-5p and CARM1 in 120 NSCLC patients were measured, and the results showed that the expression of miR-181a-5p was negatively associated with that of circHMGB2 and CARM1 level, while the expression of circHMGB2 was positively correlated with the level of CARM1 (Fig. 3M-O). Thus, circHMGB2 relieves the inhibition of the downstream molecule CARM1 by sponging miR-181a-5p in NSCLC.

### CircHMGB2 limits the efficacy of PD-1 blockade in NSCLC treatment

**Fig. 4:**
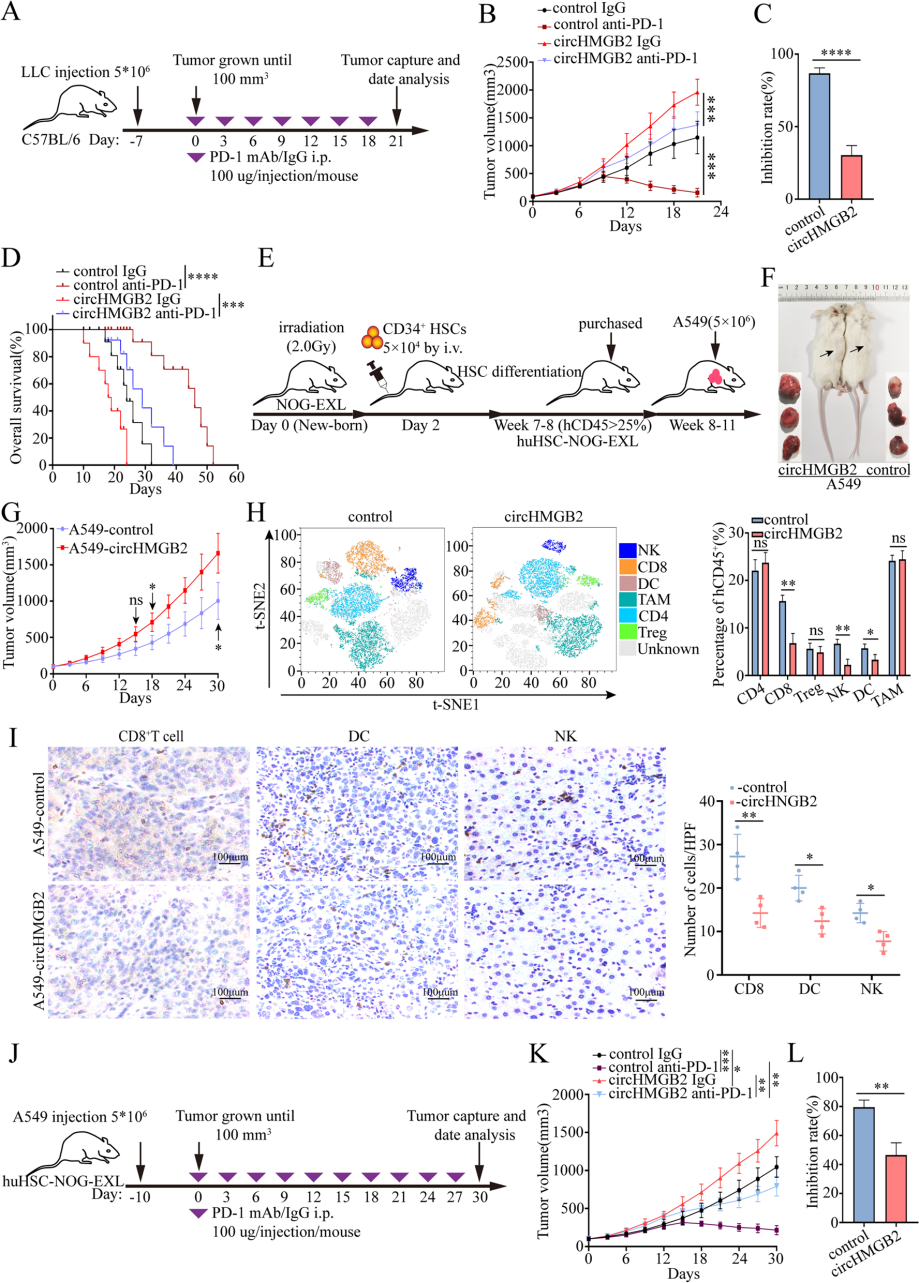
CircHMGB2 decreased the efficacy of PD-1 blockade in NSCLC treatment. **A** A schematic diagram of the treatment plan in C57BL/6 mice. **B** Growth of subcutaneous tumors in C57BL/6 mice inoculated with LLC-control or LLC-circHMGB2 cells and administered an anti-PD-1 antibody. **C** The efficacy of PD-1 therapy after the overexpression of circHMGB2. **D** The prognosis of C57BL/6 mice subcutaneously implanted with LLC-circHMGB2 cells and administered an anti-PD-1 antibody. **E** A schematic diagram of the establishment of huHSC-NOG-EXL mice. **F** and **G** huHSC-NOG-EXL mice were subcutaneously implanted with A549-control and A549-circHMGB2 cells, and the tumor size was assessed every 3 days. **H** The immune cell profiles of subcutaneous tumors derived from A549-control and A549-circHMGB2 cells were detected by flow cytometry. **I** IHC staining of CD8^+^ T cells, DCs, and NK cells in subcutaneous A549-control and A549-circHMGB2 tumors. **J** A schematic view of the treatment plan in huHSC-NOG-EXL mice. K. Growth of subcutaneous tumors in huHSC-NOG-EXL mice inoculated with A549-control or A549-circHMGB2 cells and administered an anti-PD-1 antibody. **L** The efficacy of PD-1 therapy after the overexpression of circHMGB2. Data are presented as the means ± SD; *n* = 3, *P < 0.05, **P < 0.01, ***P < 0.001, ***P < 0.0001, ns: not significant

A previous study reported that the inactivation of CARM1 could sensitize tumors to T cell-dependent immune attack [25]. Thus, we hypothesized that high expression of circHMGB2 might limit the immune response to NSCLC and inhibit the efficacy of anti-PD-1 therapy. Given the potential difference in the miR-181a-5p and CARM1 sequences between humans and mice, the predicted binding sites were compared between the two species; the results showed that humans and mice shared the same sequence of miR-181a-5p and that the predicted binding sites of Carm1 were compatible (Supplementary Fig. 5A and B). miR-181a-5p substantially decreased the luciferase activity of the wild-type Carm1 sequence but not that of the mutant Carm1 sequence (Supplementary Fig. 5C). The overexpression of circHMGB2 in LLC cells enhanced the expression of Carm1, while transfection of miR-181a-5p mimics could reverse this phenomenon (Supplementary Fig. 5D and E). Thus, circHMGB2 could also regulate the expression of the downstream molecule Carm1 in LLC cells. Moreover, the potential effects of circHMGB2 on the efficacy of anti-PD-1 treatment were evaluated in C57BL/6 mice bearing circHMGB2-overexpressing or control tumors. The PD-1 blockade or IgG was administered i.p. at a dose of 100 μg/injection every 3 days when the tumor size reached 100mm^3^ (Fig. 4A). The in vivo results showed that the overexpression of circHMGB2 significantly minimized the efficacy of anti-PD-1 treatment after 2 weeks (Fig. 4B and C). In addition, upregulated circHMGB2 expression reduced the survival of immunocompetent mice (Fig. 4D).

In order to further confirm that circHMGB2 can reshape the tumor immune microenvironment and limit the efficacy of anti-PD-1 in NSCLC, we performed in vivo experiments in mice with a humanized immune system. NOG-EXL mice can be reconstituted with human lymphoid cells and myeloid cells, and can better simulate the cellular composition of the human immune system. Consequently, these mice have been widely used in the study of tumor immunity [26]. We established huHSC-NOG-EXL mice (Fig. 4E). These mice were subcutaneously inoculated with A549-circHMGB2 and A549-control cells, and we found that circHMGB2 could dramatically potentiate the growth of subcutaneous tumors in humanized immune system mice (Fig. 4F and G). Then, the immune profiles in the tumors were assessed using flow cytometry. The results revealed the presence of exhaustion of CD8^+^ T cells, NK cells and DCs in the subcutaneous tumors derived from A549-circHMGB2 cells (Fig. 4H). The IHC staining of CD8^+^ T cells, CD56^+^ NK cells and CD11C^+^ DCs in the tumor tissues also showed a similar trend (Fig. 4I). We also verified the efficacy of anti-PD-1 treatment in humanized mice models inoculated with A549-circHMGB2 or A549-control cells, and a dosing schedule for immunotherapy was developed (Fig. 4J). The results confirmed that circHMGB2 overexpression obviously limited the efficacy of anti-PD-1 treatment (Fig. 4K and L).

Collectively, these results showed that the efficacy of anti-PD-1 treatment was limited in both a mouse model with a humanized immune system and a xenograft model of circHMGB2-overxpressing NSCLC.

### Knockout of the CARM1 gene sensitizes NSCLC cells with high circHMGB2 expression to anti-PD-1 antibody treatment

**Fig. 5:**
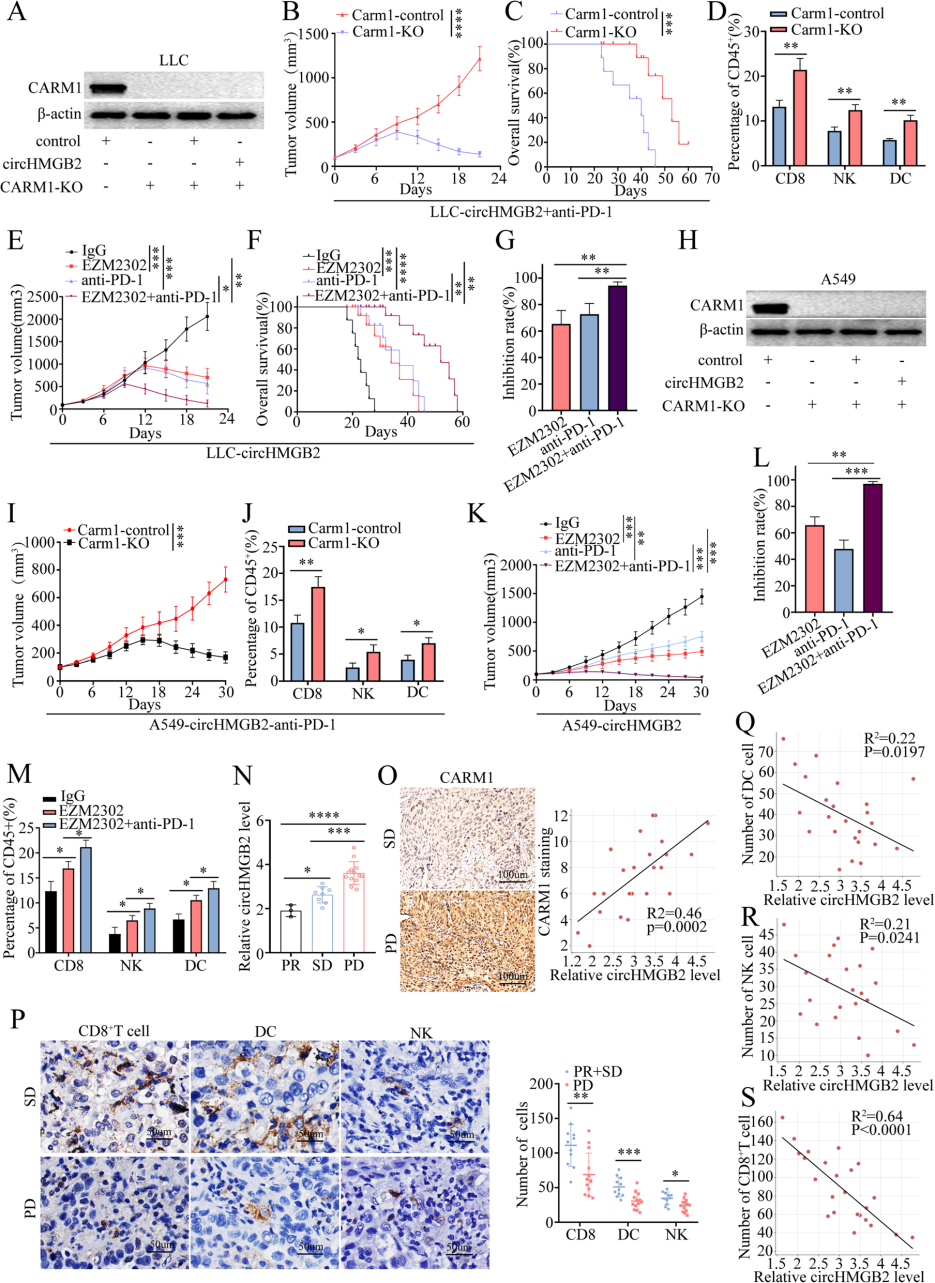
Knockout of CARM1 sensitized NSCLC cells with high expression of circHMGB2 to treatment with an anti-PD-1 antibody. **A** The knockout of the Carm1 gene in LLC cells by the CRISPR-Cas9 system was verified by western blotting. **B** The growth of subcutaneous tumors in C57BL/6 mice after the knockout of the Carm1 gene in LLC-circHMGB2 cells and the administration of an anti-PD-1 antibody. **C** The prognosis of C57BL/6 mice after subcutaneous implantation of LLC-circHMGB2 cells with Carm1 knockout and treatment with PD-1 antibody administration. **D** The infiltration of CD8^+^ T cells, NK cells and DCs in tumors overexpressing circHMGB2 and Carm1 knockout after the administration of anti-PD-1 antibody was assessed by flow cytometry. **E** The growth of subcutaneous tumors in C57BL/6 mice implanted with LLC-circHMGB2 cells after the synergistic usage of EZM2302 and an anti-PD-1 antibody. **F** The prognosis of C57BL/6 mice subcutaneously implanted with LLC-circHMGB2 cells after treatment with the synergistic combination of EZM2302 and an anti-PD-1 antibody. **G** The efficacy of the synergistic combination of EZM2302 and an anti-PD-1 antibody. **H** The knockout of the CARM1 gene in A549 cells by the CRISPR-Cas9 system was verified by western blotting. **I** The growth of subcutaneous tumors in huHSC-NOG-EXL mice after the knockout of the CARM1 gene in A549-circHMGB2 cells and the administration of an anti-PD-1 antibody. **J** The infiltration of CD8^+^ T cells, NK cells and DCs in tumors was assessed by flow cytometry which derived from A549-CARM1-control-circHMGB2 or A549-CARM1-KO-circHMGB2 cells after the administration of an anti-PD-1 antibody. **K** The efficacy of the synergistic usage of EZM2302 and an anti-PD-1 antibody in huHSC-NOG-EXL mice subcutaneously implanted with A549-circHMGB2 cells. **L** The efficacies of anti-PD-1 treatment, EZM2302 treatment or the combination anti-PD-1 and EZM2302 treatment. **M** The infiltration of CD8^+^ T cells, NK cells and DCs into tumors derived from A549-circHMGB2 cells was detected by flow cytometry after the administration of an anti-PD-1 antibody and EZM2302. **N** The expression of circHMGB2 in 24 NSCLC patients who received anti-PD-1 immunotherapy and who were stratified into three groups: the PR, SD and PD groups. **O** The relationship between CARM1 and circHMGB2 expression in NSCLC patients who received anti-PD-1 immunotherapy was assessed by IHC. **P** The infiltration of CD8^+^ T cells, DCs and NK cells in the PR + SD group and PD group who received anti-PD-1 immunotherapy. **Q**-**S** The relationship between the expression of circHMGB2 and the infiltration of CD8^+^ T cells, DCs and NK cells in 24 NSCLC patients who received anti-PD-1 immunotherapy. Data are presented as the means ± SD; *n* = 3, *P < 0.05, **P < 0.01, ***P < 0.001, ***P < 0.0001, ns: not significant, SD: stable disease, PR: partial remission, PD: progressive disease

Since the above results revealed that high circHMGB2 expression inhibited the efficacy of anti-PD-1 therapy in NSCLC, we hypothesized that the inactivation of CARM1 might sensitize the treatment of anti-PD-1. Thus, the Carm1 gene was completely knocked out with the CRISPR-Cas9 system in LLC-circHMGB2 cells (Fig. 5A). To verify the synergetic effects of Carm1 knockout and PD-1 immunotherapy, a xenograft model was established by subcutaneously implanting mice with LLC-Carm1-KO-circHMGB2 and LLC-Carm1-control-circHMGB2 cell lines. The results showed that the knockout of Carm1 significantly delayed the growth of circHMGB2-overexpressing tumors after the administration of anti-PD-1 and greatly improved the survival of the mice in this group (Fig. 5B and C). Flow cytometry analysis revealed that anti-PD-1 therapy increased the infiltration of CD8^+^ T cells, NK cells and DCs into subcutaneous circHMGB2-overexpresing tumors in C57BL/6 mice after the knockout of Carm1 (Fig. 5D). Importantly, the administration of EZM2302 (a small molecule inhibitor of CARM1) and an anti-PD-1 antibody achieved similar results (Fig. 5E-G). We knock out CARM1 gene in A549 cells (Fig. 5H), and established A549-CARM1-control-circHMGB2 and A549-CARM1-KO-circHMGB2 stably transfected cell lines. Then, the subcutaneous tumor models in humanized mice with A549-CARM1-control-circHMGB2 and A549-CARM1-KO-circHMGB2 cells were established, and those mice were administrated anti-PD-1 mAb. The in vivo experiments verified that the knockout of CARM1 significantly delayed the growth of tumors overexpressing circHMGB2 after the administration of anti-PD-1 and promoted the infiltration of CD8^+^ T cells, NK cells and DCs in the subcutaneous tumors tissue (Fig. 5I and J). Moreover, the administration of EZM2302 and anti-PD-1 mAb achieved similar results (Fig. 5K-M). The in vivo experiments showed that the EZM2302 treatment could increase the infiltration of CD8^+^ T cells, NK cells and DCs (Fig. 5M), which indicate that EZM2302 may have synergistic effect with anti PD-1 Ab to improve the anti-tumor response in murine cancer models.

To further study the expression of circHMGB2 and the efficacy of PD-1 blockade therapy, we also analyzed the expression patterns in 24 patients who received anti-PD-1 therapy. According to the RECIST1.1 standard, 13 patients achieved progressive disease (PD) status, 8 patients achieved stable disease (SD) status, and only 3 patients were considered to have achieved partial remission (PR) status. The qRT–PCR results showed that the expression of circHMGB2 in the PD group was significantly higher than that in the PR and SD groups (Fig. 5N). Spearman analysis showed that the expression of circHMGB2 was positively correlated with the expression of CARM1 in the PD, PR and SD groups (Fig. 5O). Moreover, IHC staining showed that the infiltration of CD8^+^ T cells, NK cells and DCs in the PR and SD groups was significantly higher than that in the PD group (Fig. 5P). Spearman analysis showed that the expression of circHMGB2 was positively correlated with the expression of CARM1 but negatively correlated with the infiltration of CD8^+^ T cells, DCs and NK cells in 24 NSCLC patients received anti-PD-1 therapy (Fig. 5Q-S). Thus, these data show that the inhibition of CARM1 can improve the efficacy of anti-PD-1 therapy in NSCLC patients with high circHMGB2 expression.

### CircHMGB2 inactivates the type 1 IFN response to NSCLC via CARM1

**Fig. 6:**
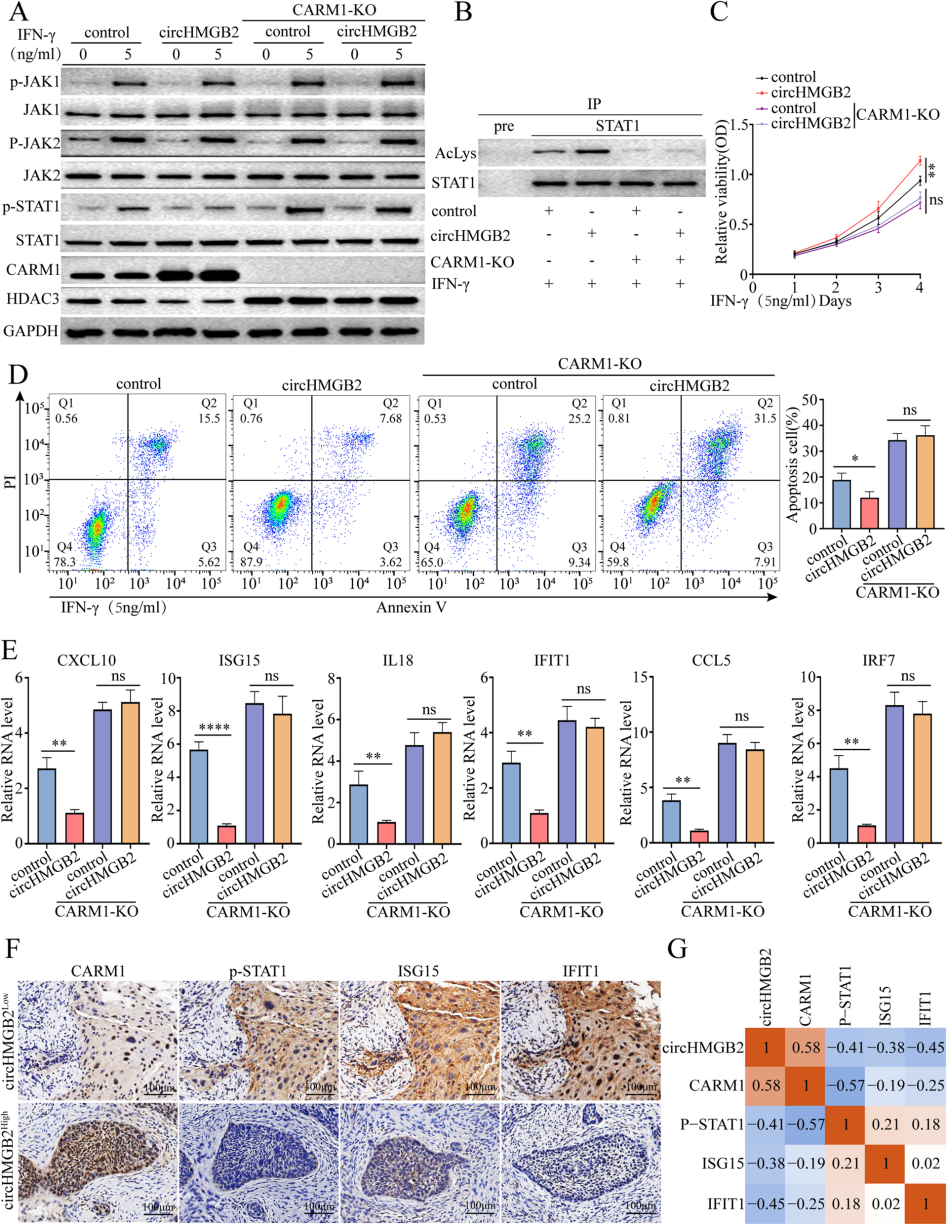
CircHMGB2 inhibited the type 1 IFN response via CARM1 in NSCLC. **A** Activation of the STAT1 pathway in A549-circHMGB2 cells after stimulation with IFN-γ for 48 h was verified by western blotting. **B** STAT1 acetylation in circHMGB2-overexpressing A549 cells or CARM1-KO A549 cells was analyzed by STAT1-IP and anti-acetyl lysine Western blotting. **C** The effect of CARM1 on the proliferation of A549-circHMGB2 cells stimulated with IFN-γ was measured by CCK-8 assay. **D** The effect of CARM1 on the apoptosis of A549-HMGB2 cells stimulated with IFN-γ was measured by flow cytometry. **E** Expression levels of type 1 IFN response-related genes were measured by qRT–PCR after the overexpression of circHMGB2 and the knockout of CARM1 in A549 cells. **F** and **G** The relationship between the expression of circHMGB2 and the staining of CARM1, p-STAT1, ISG15 and IFIT1 in 120 NSCLC patients was assessed by IHC and qRT–PCR. Data are presented as the means ± SD; *n* = 3, **P < 0.01, ***P < 0.001, ns: not significant

We had showed that the high level of circHMGB2 was associated with the exhaustion of CD8^+^ T, NK and DC cells in C57BL/6 mice and human NSCLC samples. Moreover, elevated circHMGB2 was identified to upregulate the expression of the downstream molecule CARM1 by sponging miR-181a-5p, which indicated that high level of circHMGB2 could reset the tumor immune microenvironment by CARM1. Coincidently, a previous study identified that CARM1 could inhibit the type 1 IFN response in tumors and desensitize tumors to the cytotoxic T cell-mediated immune response [25]. Thus, we hypothesized that high expression of circHMGB2 might inactivate the type 1 IFN response via CARM1 to enhance NSCLC resistance to cytotoxic T cells and foster an immunosuppressive environment in NSCLC. The knockout of the CARM1 gene in A549-control or A549-circHMGB2 cells also led to a significantly increased response to IFN-γ compared to that of A549-circHMGB2 cells. Mechanistically, stimulation with IFN-γ (5 ng/ml) upregulated the expression of IFN-activated genes and activated JAK and STAT1, which are involved in a pathway that is important for the type 1 IFN response (Fig. 6A) [27, 28]. In order to further demonstrate the mechanism of CRAM1 in IFN-γ signal, Co-IP combined with MS was used employed to isolate and identify the interactome of CRAM1 in A549, PC9 and H1299 cells, and HDAC3 was found and further identified to interact with CRAM1(Supplementary Fig. 6A and B). HDAC3 levels did not increase after stimulation with IFN-γ; however, overexpression of circHMGB2 induced a reduction in the HDAC3 levels. Moreover, CARM1 KO significantly increased HDAC3 expression (Fig. 6A). HDAC3 is involved in the regulation of the balance between STAT1 phosphorylation and acetylation [29]. In Co-IP experiments, overexpression of circHMGB2 inhibited STAT1 deacetylation, and CARM1 KO reversed this phenomenon (Fig. 6B). These findings confirmed that overexpression of circHMGB2 inhibited STAT1 deacetylation, thereby reducing the phosphorylation of STAT1 and inhibiting IFN-γ signal transduction. The proliferation of CARM1-knockout A549 cells was significantly impaired compared to that of A549-circHMGB2 or control cells, while the apoptosis of CARM1-KO A549 cells was remarkably enhanced after stimulation with IFN-γ (Fig. 6C and D). In addition, the expression of IFN-activated genes, including CXCL10, ISG15, IL18, IFIT1, CCL5 and IFR7, was measured using qRT–PCR. The results showed that the expression of these genes were significantly increased after the knockout of CARM1 but inhibited after the overexpression of circHMGB2 (Fig. 6E, Supplementary Fig. 6C). To further verify the role of circHMGB2 in regulating the type 1 IFN response, the phosphorylation of STAT1, the expression of CARM1 and the expression of the IFN response genes ISG15 and IFIT were evaluated in tumor tissues from 120 NSCLC patients using IHC. The results suggested that the expression of circHMGB2 was positively correlated with the staining of CARM1 but negatively correlated with the phosphorylation of STAT1 and the expression of ISG15 and IFIT (Fig. 6F and G). Thus, these results indicate that circHMGB2 suppresses the type 1 IFN response via CARM1, which induces resistance to the cytotoxic immune response in NSCLC.

**Fig. 7:**
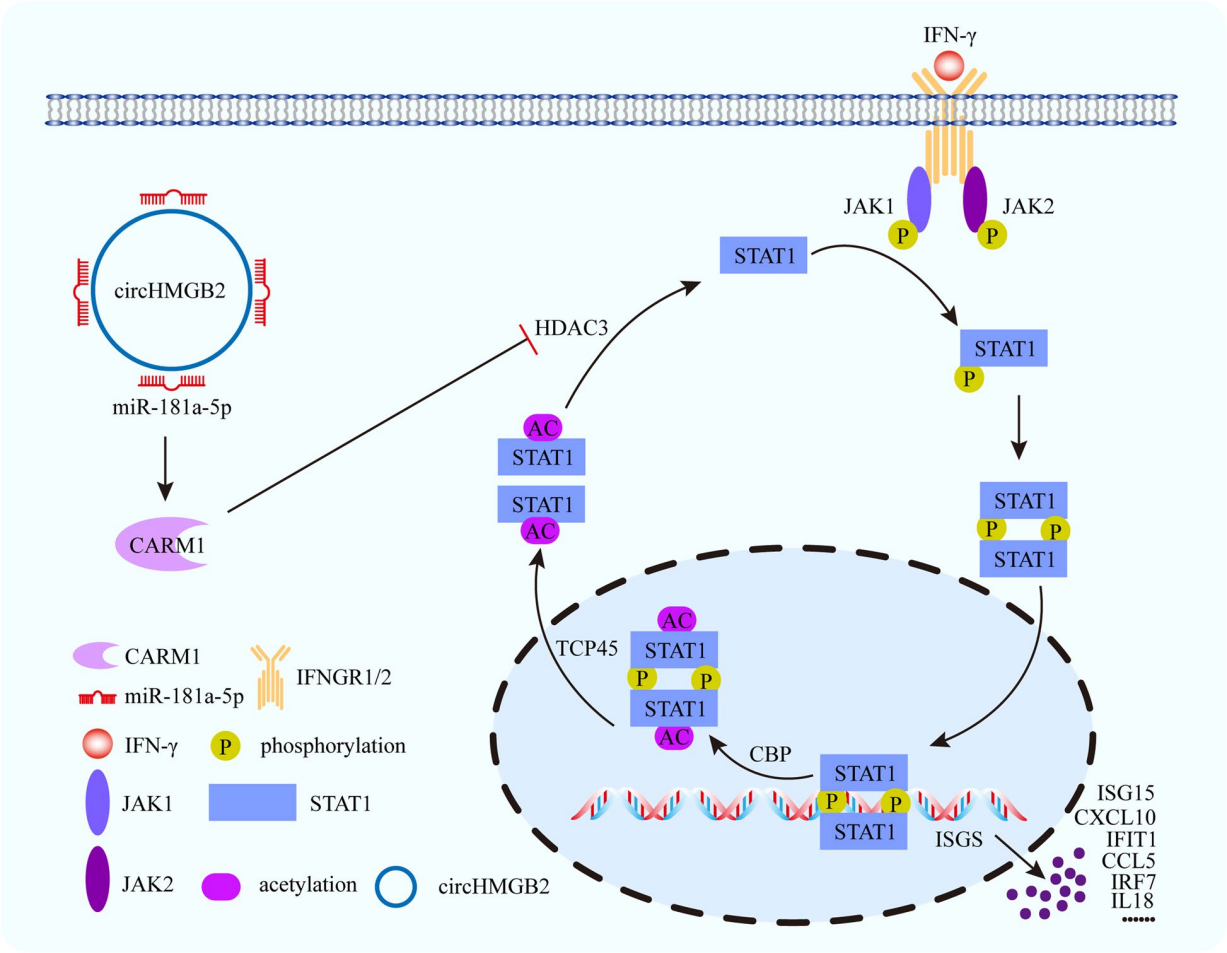
Schematic diagram illustrates the mechanism of circHMGB2 relieves the inhibition of the downstream molecule CARM1 by sponging miR-181a-5p, which induces a reduction in the HDAC3 levels. Then, STAT1 deacetylation is inhibited, thereby reducing the phosphorylation of STAT1 and inhibiting IFN-γ signal transduction. Subsequently, the expression of IFN-activated genes, including CXCL10, ISG15, IL18, IFIT1, CCL5 and IFR7, were inhibited. Those drive immunosuppression and anti-PD-1 resistance in lung adenocarcinomas and squamous cell carcinomas

Figure 7 summarizes the main findings of this study. In NSCLC, the upregulation of circHMGB2 expression shapes the immunosuppressive microenvironment and leads to resistance to immunotherapy. Mechanistically, circHMGB2 relieves the inhibition of the downstream molecule CARM1 by sponging miR-181a-5p, which inhibits the type 1 IFN response by HDAC3 and promotes resistance to cytotoxic T cells. In addition, we found that the synergistic combination of EZM2302 and anti-PD-1 treatment substantially increased the susceptibility to immunotherapy in NSCLC patients with high circHMGB2 expression.

## Discussion

Since circRNAs were first discovered in the cytoplasm of eukaryotic cells in 1976, various circRNAs have been identified by high-throughput deep sequencing [30, 31]. Previous studies have shown the differential expression of many circRNAs in various cells and reported the potential biological functions of circRNAs in various pathophysiological processes, such as cancer, heart failure, atherosclerosis and immunity [32–35]. In addition, abnormally expressed circRNAs are closely related to cancer development, progression and treatment resistance. However, the underlying molecular mechanisms are still unclear [35, 36].

Previous studies have confirmed the oncogenic role of HMGB2 in various cancers. After the detection of 3 HMGB2-derived circRNAs in 8 pairs of NSCLC tissues and matched normal tissues, circHMGB2 (hsa_circ_0071452) was found to be highly expressed in tumor tissues, and high circHMGB2 expression was identified as an indicator of poor prognosis in NSCLC patients. Moreover, we further showed that circHMGB2 not only modulated the proliferation of NSCLC cells but also restricted the immune response by fostering the immunosuppressive cancer microenvironment. Notably, the overexpression of circHMGB2 was found to inhibit the type 1 IFN response in NSCLC and enhance the exhaustion of cytotoxic T cells via the miR-181a-5p/CARM1 axis. Moreover, the synergistic combination of the CARM1 inhibitor EZM2302 and an anti-PD-1 antibody significantly inhibited the growth of circHMGB2-overexpressing tumors in mice with a humanized immune system and in immunocompetent mice; these results indicated a promising strategy for immunotherapy for the subgroup of NSCLC patients with high circHMGB2 expression. Therefore, this study provides a new oncogene that induces the formation of an immunosuppressive microenvironment and impairs the efficacy of anti-PD-1 therapy in NSCLC.

The biological functions of circRNAs have been thoroughly investigated in various cancers. Competitive endogenous RNAs (ceRNAs) are a unique feature of circRNAs and have been widely reported in numerous studies [6]. CircRNAs contain nucleotide sequences that interact with miRNAs, and the sponging of miRNAs by circRNAs interrupts the binding of miRNAs to the 3′-UTR regions of target mRNAs, thus eliminating the inhibition of downstream mRNAs and regulating the biological function of cancers [37]. In addition, circRNAs can interact with RNA-binding proteins, which are vital for the transcription and translation of genes or encoded proteins that regulate the progression of cancers [38, 39]. Moreover, circRNAs can directly regulate the transcription of genes, bypassing the sponging of miRNAs [40]. In this study, circHMGB2 was shown to function as a ceRNA for miR-181a-5p and further induce the upregulation of the downstream molecule CARM1. Thus, we conclude that the circHMGB2/miR-181a-5p/CARM1 axis reshapes the TME in NSCLC.

The communication network between cancer cells and the TME is intricate, and understanding the underlying mechanisms is vital to preventing immune evasion and developing effective therapeutic strategies. Previous studies have confirmed the critical roles of circRNAs in the regulation of tumor immunity by both enhancing and inhibiting antitumor immunity [41]. Mechanistically, circRNAs can regulate the expression of immune-related genes in cancer cells at the posttranscriptional level and impact the TME. For instance, circRNAs can modulate the expression of molecules related to the immune checkpoint PD-1/PD-L1 axis and influence the cytotoxic function of effector T lymphocytes [42]. In addition, since exosomes and extracellular vesicles facilitate communication between cells, circRNAs can be transmitted from host cells to the TME via these vehicles to regulate biological functions in immune cells [37]. Furthermore, the levels of circRNAs in the plasma of cancer patients reflect immune infiltration into the TME [43]. In this study, we found that circHMGB2 had a slight effect on the proliferation, but not on the invasion, of NSCLC; it inhibited IFN response-related gene expression and enhanced tumor resistance to cytotoxic T cells, further inducing an immunosuppressive microenvironment in NSCLC.

The type 1 IFN response was first shown to interfere with the antiviral immune response, which protects the host from a second virus attack after primary viral infection [44]. In addition to its antiviral effects, accumulating evidence has confirmed that the type 1 IFN response enhances host immunity and plays a critical role in modulating the activation, differentiation and apoptosis of various subgroups of immune cells [45]. Moreover, previous studies showed the potential effects of type 1 IFNs on host immunosurveillance in the TME and antitumor therapy. The efficacy of antitumor chemotherapy, radiotherapy, targeted therapy and immunotherapy greatly depends on the activation of the type 1 IFN response [44, 46]. In this study, the overexpression of circHMGB2 inhibited the type 1 IFN response and decreased the efficacy of anti-PD-1 therapy in NSCLC. The transcriptional coactivator CARM1 has been reported to be widely involved in biological processes such as cell differentiation, autophagy, metabolism and cancer [47]. Recently, CARM1 was discovered to be a negative regulator of tumor immunity that contributes to resistance to checkpoint blockade therapy [25]. Thus, we found that circHMGB2 induces the formation of an immunosuppressive TME via the miR-181a-5p/CARM1 axis, which inhibits the type 1 IFN response in cancer cells and enhances cancer cell resistance to the cytotoxic effects of T cells.

The discovery of the immune checkpoint PD-1 was a breakthrough in cancer immunotherapy, and drugs targeting this checkpoint have been rapidly commercialized in recent years. Interruption of the PD-1/PD-L1 axis reverses the dysfunctional status of cytotoxic T cells and enhances the immune surveillance of cancer cells. Nonetheless, a large number of patients have no obvious response to PD-1 blockade, which is attributed to the complicated biological regulatory network in most advanced cancers [48]. Therefore, an increasing number of studies have investigated the mechanism underlying resistance to anti-PD-1 therapy and explored the dual blockade of the PD-1/PD-L1 axis and other targets that led to the failure of PD-1 blockade [49]. Since circHMGB2 overexpression upregulated the expression of the downstream target CARM1, which facilitated the formation of an immunosuppressive TME and decreased the efficacy of anti-PD-1 monotherapy, we explored the feasibility of the dual blockade of PD-1 and CARM1. The preclinical results revealed that EZM2302 evidently improved the efficacy of PD-1 blockade in circHMGB2-overexpressing NSCLC in a synergistic manner.

## Conclusion

This study revealed a critical role of circHMGB2 in the TME of LUAD and LUSC, and provided a new strategy for improving the efficacy of PD-1 immunotherapy in LUAD and LUSC.

## Supplementary Information

**Additional file 1.**

**Additional file 2: Supplementary Fig. 1.** A. The expression of circUSP7 was measured in 3 pairs of NSCLC tissues and matched normal tissues. CircUSP7 was used as a positive control in this study. B. The expression of circHMGB2 was analyzed according to tumor diameter (< 2 cm vs. ≥ 2 cm), lymph node metastasis status (yes vs. no), and TNM stage in 78 LUAD patients. C, Survival analysis of the recurrence and OS of 78 LUAD patients divided into groups according to circHMGB2 expression (circHMGB2^high^ vs. circHMBG2^low^) was performed using Kaplan–Meier and log rank analysis. Data are presented as the means ± SD; *n* = 3, *P < 0.05, **P < 0.01, ***P < 0.0001. **Supplementary Fig. 2.** A. The expression of circHMGB2 was measured in HBE, LLC and 5 NSCLC cell lines (NCI-H460, A549, PC9, H1703, and NCI-H1299 cells) using qRT–PCR. B. The transfection efficiency of three stable cell lines, A549-circHMGB2, H1299-shcircHMGB2 and LLC-circHMGB2, was validated by qRT–PCR. C. The invasion of A549-circHMGB2 and H1299-shcircHMGB2 cells was assessed by Matrigel Transwell assay. D. The migration of A549-circHMGB2 and H1299-shcircHMGB2 cells was assessed by wound healing assay. E, IHC staining of CD4^+^ T cells, Tregs, and TAM in subcutaneous tumors derived from LLC-control and LLC-circHMGB2 cells. Data are presented as the means ± SD; *n* = 3, ****P < 0.0001, ns: not significant. **Supplementary Fig. 3.** A. Electron microscopy image of exosomes in the supernatants of H460 cells. B. The levels of biomarkers of exosomes from the supernatants of NSCLC cells were measured by western blotting. C. The expression of circHMGB2 in exosomes derived from the supernatants of H460-control and H460-circHMGB2 cells was measured by qRT–PCR. Data are presented as the means ± SD; *n* = 3, ns: not significant. **Supplementary Fig. 4.** A. The RNA pulldown assay was performed with PC9, A549 and H1299 cells transfected with biotinylated miR-181a-5p. B, The luciferase activity of SIRT1 was measured in HEK-293 T cells transfected with miR-181a-5p. C. The luciferase activity of CDK8 was measured in HEK-293 T cells transfected with miR-181a-5p. D. The luciferase activity of IRS2 was measured in HEK-293 T cells transfected with miR-181a-5p. E. The luciferase activity of BHLHE40 was measured in HEK-293 T cells transfected with miR-181a-5p. Data are presented as the means ± SD; *n* = 3, *P < 0.05, **P < 0.01, ***P < 0.001, ***P < 0.0001, ns: not significant. **Supplementary Fig. 5.** A. Comparison of the nucleotide sequences between hsa-miR-181a-5p and mmu-miR-181a-5p. B. The putative binding sites of murine Carm1 and miR-181a-5p. C. The luciferase activity of Carm1 was measured in HEK-293 T cells transfected with miR-181-5p. D. The expression of Carm1 was measured in LLC cells overexpressing both circHMGB2 and miR-181a-5p using qRT–PCR. E. The expression of Carm1 was measured in LLC cells overexpressing both circHMGB2 and miR-181a-5p using western blotting. Data are presented as the means ± SD; *n* = 3, ****P < 0.0001, ns: not significant. **Supplementary Fig. 6.** A, MS (mass spectrometry) was performed in PC9, A549 and H1299 cells respectively. B, The Co-IP assay was performed in A549 cells to confirm the interaction between CARM1 and HDAC3. C, The expression of type 1 IFN response-related genes was measured in circHMGB2-overexpressing or CARM1-knockout LLC cells after stimulation with IFN-γ for 48 h. Data are presented as the means ± SD; *n* = 3, ***P < 0.001, ***P < 0.0001, ns: not significant.

## Data Availability

All data in our study are available upon request.

## Abbreviations

NSCLC: non-small-cell lung cancer
LUAD: lung adenocarcinomas
LUSC: squamous cell carcinomas
circRNA: circular RNA
HMGB2: high-mobility group box 2
qRT–PCR: quantitative real-time polymerase chain reaction
PD-1: programmed cell death 1
TMA: tissue microarray
IHC: immunohistochemistry
FISH: fluorescence in situ hybridization
CCK-8: cell counting kit-8
circRIP: circRNA precipitation
miRNA: microRNA
NC: negative control
OS: overall survival
RFS: recurrence-free survival
TME: tumor microenvironment
PD: progressive disease
SD: stable disease
PR: partial remission
