# Supplementary material for: The circular RNA circHMGB2 drives immunosuppression and anti-PD-1 resistance in lung adenocarcinomas and squamous cell carcinomas via the miR-181a-5p/CARM1 axis
Source: Mol Cancer. 2022 May 7;21:110. doi: 10.1186/s12943-022-01586-w (PMC9077876; doi:10.1186/s12943-022-01586-w)
Supplement: Supplementary file 1 — Additional file 1. [file 12943_2022_1586_MOESM1_ESM.docx]

**Supplementary Materials and Methods**

**Agarose gel electrophoresis**

Agarose gel electrophoresis is used to analyze the size of PCR products. 200 ml 1×TAE buffer and 3g agarose powder（Beyotime, China）were added into the Erlenmeyer flask, and were heated until the agarose was completely dissolved. Then 20μl GelRed (Tsingke, Beijing, China) was added and mixed. The agarose solution was poured into the mold prepared in advance and cooled to room temperature. Remove the comb, and put the gel into the electrophoresis tank. Then the 1×TAE buffer was added until covering 5mm above the gel. 5μl PCR production to be detected and 1μl 6×Loading buffer (Tsingke) were mixed. Subsequently, 5μl DNA Ladder (Tsingke) and 6μl PCR sample were added separately to the sample wells. Start electrophoresis at 100V for 1h after samples loading. After the electrophoresis was completed, the gel was detected in a Gel imaging system (Tanon, China) and the results were analyzed.

**Western blot**

The cells were washed with PBS for three times and lysed by RIPA buffer (Yesen, China). Then, the total protein was separated via electrophoresis and transferred onto PVDF membranes (Millipore, USA). After incubation with protein free rapid blocking buffer for 15min (EpiZyme, China), the membranes were submerged in the primary antibody at 4°C for 12h. After washed with TBST for three times, the membranes were reacted with the secondary antibody for 1h. Finally, the targeted antibody was detected with ECL in a chemiluminescence imaging system (Tanon, China).

**qRT-PCR**

The cells were washed with PBS for three times and lysed by TRIzol (Invitrogen, USA) to extract the total RNA. Then, the concentration of total RNA was measured by Drop 2000 (Thermo Fisher) and it was reversely transcripted into cDNA according to the instructions of the manufacturer’s kit (Yesen, China). Next, the RT-qPCR was performed on the Applied Biosystem with the reaction volume of 10 µl. The gene GAPDH was used as an internal parameter for circRNAs and mRNA, and U6 snRNA was used as a reference for miRNA.

**IHC**

IHC staining of the target proteins was performed on TMAs. The primary antibodies used in this article were described as following: monoclonal mouse anti-human CD8 (dilution 1:4000, Proteintech, China); monoclonal rabbit anti-mouse CD8 (dilution 1:1000, CST, USA); rabbit monoclonal anti-Foxp3 (dilution 1:100, Abcam, USA); rabbit monoclonal anti-56 (dilution 1:100, Abcam, USA); monoclonal rabbit anti-CD161(dilution 1:100, Abcam, USA); Polyclonal rabbit anti- CD11C (dilution 1:1000, Proteintech, China); monoclonal rabbit anti-CD4 (dilution 1:400, Beyotime, China); Polyclonal rabbit anti-mouse CD68 (dilution 1:1000, Abcam, USA); monoclonal rabbit anti-human CARM1 (dilution 1:500, Abcam, USA); Polyclonal rabbit anti-p-STAT1 (dilution 1:100, Abcam, USA); Polyclonal rabbit anti-ISG15 (dilution 1:200, Abclonal, China); Rabbit anti-IFIT1 polyclonal antibody (dilution 1:200, absin, China). For the evaluation on the staining of p-STAT1, CARM1, ISG15 and IFIT1, the Image-Pro Plus version 6.2 software (Media Cybernetics) was used, which gave the final scores based on the density and intensity of the positive staining. In addition, the evaluation on the infiltration of CD8, CD4, Treg, DCs, NK and TAM in the TMA, the positive cells were counted under 4 areas of 200× images. The average positive cells were counted as the final score.

**Fluorescence in situ hybridization**

The signals of circHMGB2 and miR-181a-5p were detected using a FISH kit (GenePharma) according to the manufacturer’s instruction. The images were pictured by the fluorescence microscope. The sequence on the probe of circHMGB2 and miR-181a-5p were presented in supplementary table 3.

**Wound healing assay**

After the density of cells in the 6-well plates reached over 90%, a line was made across the middle of the plate via a pipette tip. After being washed with PBS for three times, the plates were pictured with a microscope and repeated to observe every 24h. The covered area was calculated with ImageJ software.

**Matrigel Transwell assay**

The Matrigel transwell assay was performed in the 8.0-μm-pore Transwell plates precoated with 0.5 µg/µl Matrigel. A total of 1×10^5^ cells were mingled with serum-free medium and added into the upper chamber, while another 500µl medium with 10% FBS was added into the lower chamber. After 48h. the chambers were fixed with 4% paraformaldehyde for 10min and then dyed by 0.4% crystal violet for 15 minutes. The cells on the pore were pictured with a microscope and the number of cells was calculated with ImageJ.

**CCK-8 and colony formation assays**

The CCK-8 and colony formation assays were used to measure the proliferation of cells.

The CCK-8 assay was performed on 96-well plates. Every well was seeded with 1000 cells and continue to culture for 10h. Then, 10µl CCK-8 solution was added to the well and further incubated for 90min. Next, the absorbance in 450nm was measured every 24h, which lasted for 4 days. The colony formation assay was performed on 6-well plates. After cell counting, 1000 cells were seeded into the plate with 3ml medium with 10%FBS and cultured for 14 days. Finally, the cells were fixed with 4% paraformaldehyde for 10 minutes and dyed by 0.4% crystal violet for 15 minutes.

Supplementary table 1 Antibody for western blotting, RIP, Co-IP, and immunohistochemistry

| **Antibody** | **Company** | **Cat No.** |
| --- | --- | --- |
| CD8(mouse anti-human) | Proteintech | 66868-1-Ig |
| CD56 | CST | 99746s |
| CD161/NK1.1 | Abcam | ab234107 |
| CD11C | Proteintech | 17342-1-AP |
| CD4 | Beyotime | AF6393 |
| CD68 | Abcam | ab125212 |
| Foxp3 | Abcam | Ab215206 |
| CARM1 | Abcam | ab243638 |
| JAK1 | Abcam | ab133666 |
| P-JAK1 | CST | 74129S |
| JAK2 | Abcam | ab108596 |
| P-JAK2 | Abcam | ab32101 |
| P-STAT1 | Abcam | ab30645 |
| STAT1 | Abcam | ab239360 |
| ISG15 | ABclonal | A1182 |
| IFIT1 | absin | abs149003 |
| β-actin | YEASEN | 30101ES50 |
| HDAC3 | Abcam | Ab32369 |
| PD-1 | Abcam | ab214421、ab52587 |
| Acetylated-Lysine Antibody | CST | 9441S |
| Peroxidase-Conjugated Goat Anti-Rabbit IgG(H+L) | YEASEN | 33101ES60 |
| Peroxidase AffiniPure Goat Anti-Mouse IgG (H+L) | YEASEN | 33201ES60 |
| AGO2 | Abcam | ab32381 |
| IgG | Abcam | ab172730 |
| Brilliant Violet 711™ anti-mouse CD45 Antibody | BioLegend | 103147 |
| FITC anti-mouse CD3 Antibody | BioLegend | 100203 |
| Brilliant Violet 510™ anti-mouse CD4 Antibody | BioLegend | 100559 |
| Brilliant Violet 605™ anti-mouse CD8a Antibody | BioLegend | 100744 |
| APC/Cyanine7 anti-mouse/human CD11b Antibody | BioLegend | 101226 |
| APC anti-mouse CD11c Antibody | BioLegend | 117309 |
| PE/Cyanine7 anti-mouse F4/80 Antibody | BioLegend | 123113 |
| PE/Dazzle™ 594 anti-mouse NK-1.1 Antibody | BioLegend | 108747 |
| PE anti-mouse FOXP3 | BioLegend | 126404 |
| TruStain FcX™ (anti-mouse CD16/32) Antibody | BioLegend | 101319 |
| Brilliant Violet 711™ anti-human CD45 | BioLegend | [304049](http://bioec.cn/r/304049-BLG) |
| FITC anti-human CD3 Antibody | BioLegend | 300405 |
| Brilliant Violet 510™ anti-human CD4 Antibody | BioLegend | 317443 |
| APC anti-human CD11c Antibody | BioLegend | 301613 |
| Brilliant Violet 605™ anti-human CD8a Antibody | BioLegend | 301039 |
| PE/Cyanine7 anti-human CD68 Antibody | BioLegend | 333815 |
| PE/Dazzle™ 594 anti-human CD56 Antibody | BioLegend | 318347 |
| PE anti-human FOXP3 | BioLegend | 320107 |
| Human TruStain FcX™ | BioLegend | 422301 |
| CD8(rabbit anti-mouse) | CST | 98941T |
| GAPDH | Beyotime | AF0006 |
| TSG101 | Abcam | ab133586 |
| CD63 | Abcam | ab271286 |
| GM130 | Abcam | ab52649 |

Supplementary table 2 The qRT-PCR primers used in this study

| **Gene** | **Forward primer (5’-3’)** | **Reverse primer (5’-3’)** |
| --- | --- | --- |
| CARM1 | ATCGCCCTCTACAGCCATGA | GTACTGCACGGCAGAAGACT |
| Carm1 | CCGGATCCTGATGGCCAAAT | GCAACATCGAACCAGAAGGC |
| HMGB2 | TGAGGAAAAGCTCGCACCAG | TTGCAGACATGGTCTTCCATC |
| β-actin | GAGCACAGAGCCTCGCCTTT | TCATCATCCATGGTGAGCTGG |
| GAPDH | AGGTCGGAGTCAACGGATTTG | TGACAAGCTTCCCGTTCTCA |
| Gapdh | CCCAGCTTAGGTTCATCAGGT | CCAATACGGCCAAATCCGTT |
| CXCL10 | CCACGTGTTGAGATCATTGCT | TGCATCGATTTTGCTCCCCT |
| ISG15 | ACAGCCATGGGCTGGGA | GTTCGTCGCATTTGTCCACC |
| IL18 | CGCAGATGGCTCTTTGCTTT | CGCAGATGGCTCTTTGCTTT |
| IFIT1 | ATTTACAGCAACCATGAGTACAAA | ATTTACAGCAACCATGAGTACAAA |
| CCL5 | GGATCAAGACAGCACGTGGA | GGATCAAGACAGCACGTGGA |
| IRF7 | TTGGCTCCTGAGAGGGCA | TTATCCCGCAGCATCACGAA |
| circUSP7 | CCAGTGTAAAGAAGTAGACTATCGG | ACGAATCATCCCCCTCGGTT |
| U6 | CTCGCTTCGGCAGCACA | AACGCTTCACGAATTTGCGT |
| **Divergent primer** |  |  |
| has_circ_0071452 | ACATGTTGAACTCAGTGAACTTTAT | TTCACTTTTGATCTTTGGGCG |
| has_circ_0071453 | AGTTTTGTACTTGACGTTTCTGTT | TTTTCCTCAGAGTCCCGCAG |
| has_circ_0071454 | TGGGTGAAATGTGGTCTGAGC | TCCCCAATGGATAGGCCAGG |
| **Convergent primer** |  |  |
| has_circ_0071452 | TTTTGCTCTGAACATCGCCC | TTCCTGCTTCACTTTTGCCC |

Supplementary table 3 The probe sequence for circHMGB2 and miR-181a-5p

| **Probe** | **Sequence** |
| --- | --- |
| circHMGB2 | CAGGAAGAAGGCAGATATAT |
| miR-181a-5p | ACTCACCGACAGCGTTGAATGTT |
| circUSP7 | TCGGTTGGCATCATGTACACAGCTATTTTTC |

Supplementary table 4 The sequence of shcircHMGB2

| **shRNA** | **Target sequence** |
| --- | --- |
| shRNA -1 | ATATATATCTGCCTTCTTCCT |
| shRNA -2 | TATATATATCTGCCTTCTTCC |
| shRNA -3 | TATATATCTGCCTTCTTCCTG |

Supplementary table 5 The Crisper Cas9 gRNA sequences

| **Target genes** | **Sequences** | **Target location** |
| --- | --- | --- |
| Mouse *Carm1* #1 | GGTTCTGCAGGATCGCACGC | Exon 4 |
| Mouse *Carm1* #2 | AAAAAGTACCTGAAGCCTAG | Exon 6 |
| Mouse *Carm1* #3 | GGCGCCCGCCTCCTCACTAT | Exon 2 |
| Mouse *Carm1* #4 | ATAGTGAGGAGGCGGGCGCC | Exon 2 |
| Human *CARM1* #1 | AGCACGGAAAATCTACGCGG | N/A |
| Human *CARM1* #2 | AACACCGACACGGTAGCGCA | Exon 2 |
| Human *CARM1* #3 | ATGCAGGACTACGTGCGGAC | Exon 3 |
| Human *CARM1* #4 | TTGCAGGATGGCGCGCTGGT | Exon 3 |
