# Supplementary material for: The circular RNA circHMGB2 drives immunosuppression and anti-PD-1 resistance in lung adenocarcinomas and squamous cell carcinomas via the miR-181a-5p/CARM1 axis
Source: Mol Cancer. 2022 May 7;21:110. doi: 10.1186/s12943-022-01586-w (PMC9077876; doi:10.1186/s12943-022-01586-w)

Figure S1

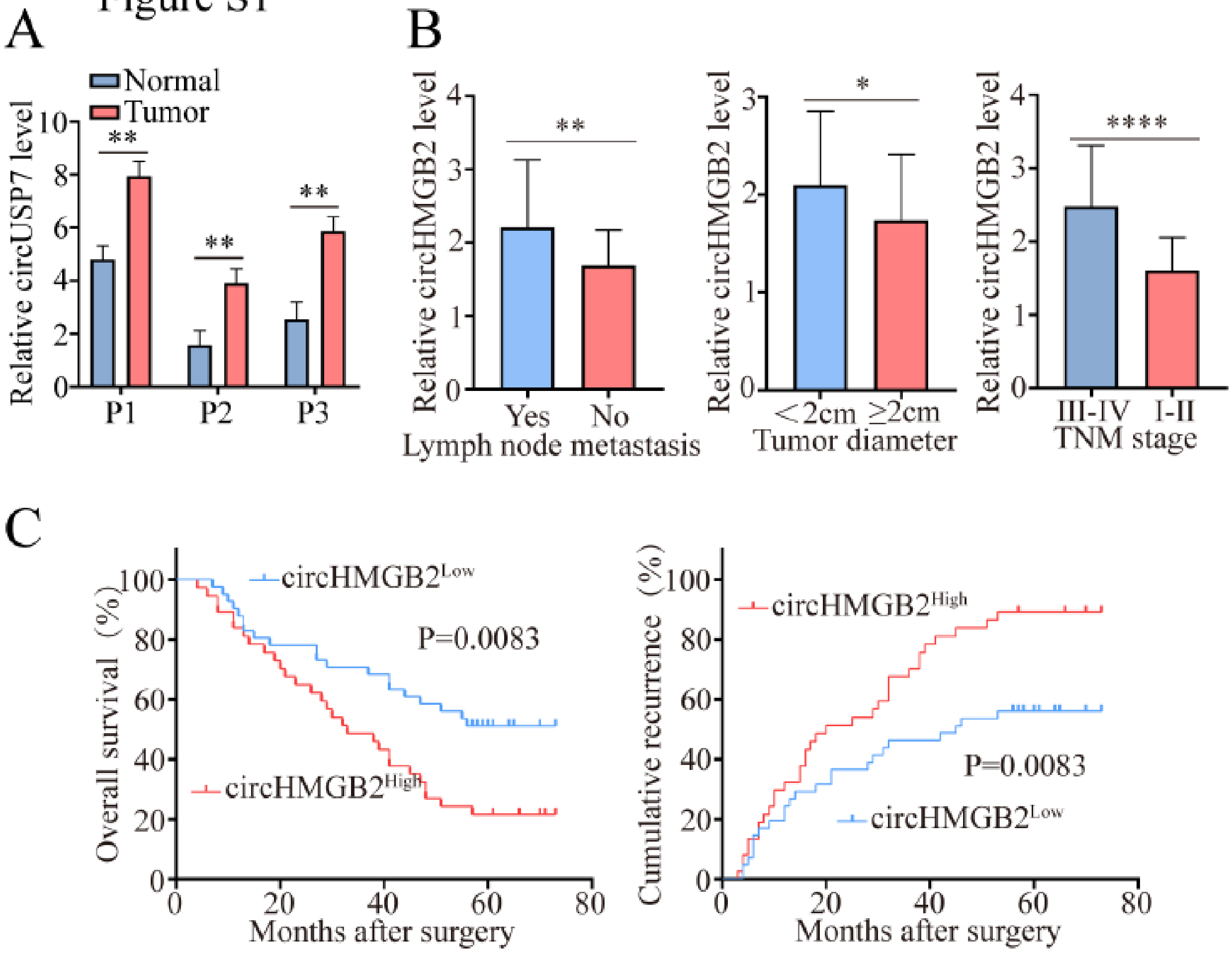
A
Normal
Tumor
Relative circUSP7 level
0
2
4
6
8
10
**
**
**
P1
P2
P3
B
Relative circHMGB2 level
**
Yes
No
Lymph node metastasis
*
＜2cm
≥2cm
Tumor diameter
****
III-IV
I-II
TNM stage
C
Overall survival (%)
circHMGB2^Low
circHMGB2^High
P=0.0083
Months after surgery
Cumulative recurrence (%)
circHMGB2^High
circHMGB2^Low
P=0.0083
Months after surgery

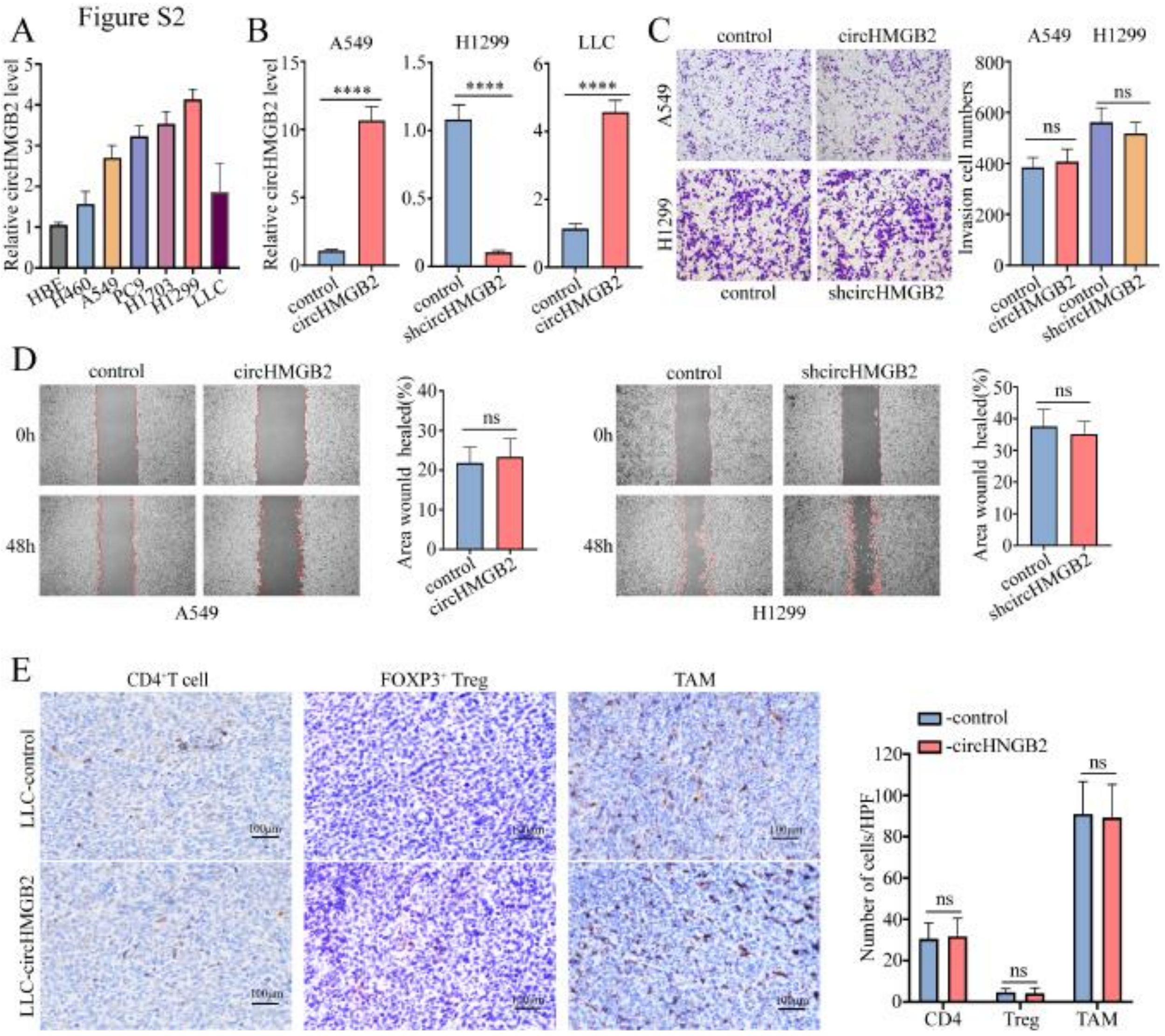
Figure S2
A
Relative circHMGB2 level
HBE
H460
A549
PC9
H1703
H1299
LLC
B
A549
H1299
LLC
Relative circHMGB2 level
****
control
circHMGB2
shcircHMGB2
C
control
circHMGB2
shcircHMGB2
A549
H1299
Invasion cell numbers
ns
D
control
circHMGB2
shcircHMGB2
0h
48h
A549
H1299
Area wounld healed(%)
ns
E
CD4+T cell
FOXP3+ Treg
TAM
LLC-control
LLC-circHMGB2
-control
-circHNGB2
Number of cells/HPF
CD4
Treg
TAM
ns

Figure S3

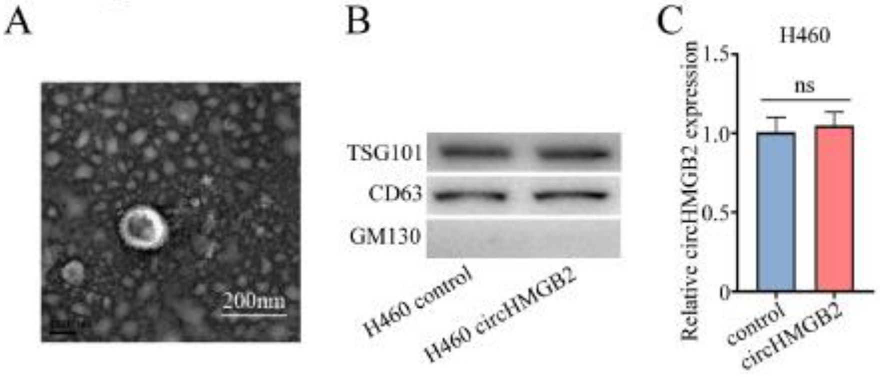
A
200nm
B
TSG101
CD63
GM130
H460 control
H460 circHMGB2
C
H460
Relative circHMGB2 expression
1.5
1.0
0.5
0
ns
control
circHMGB2

Figure S4

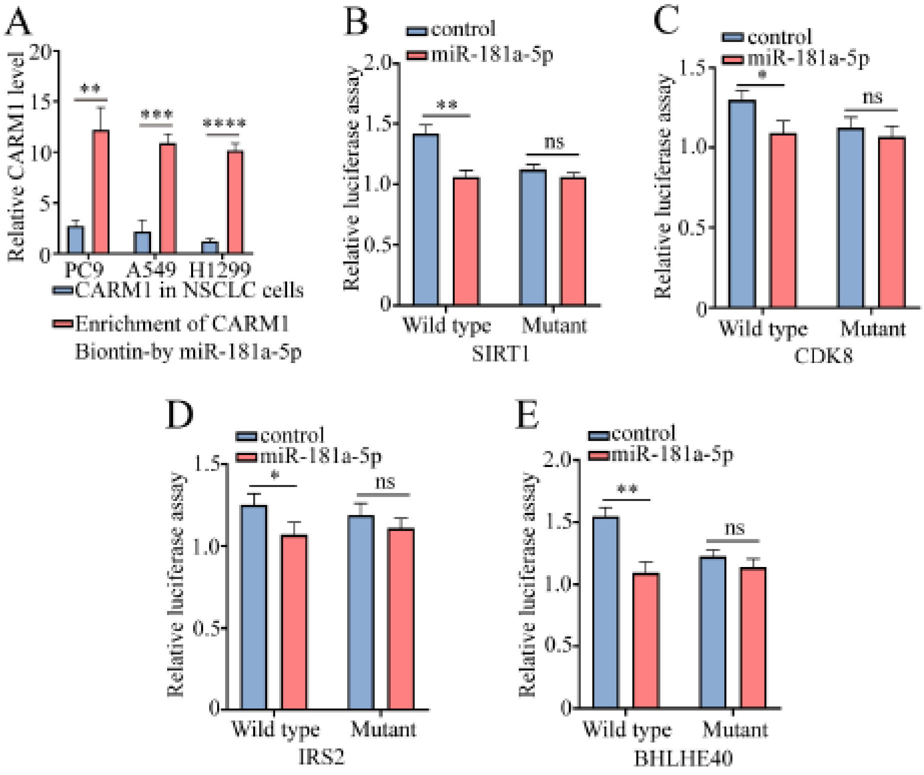
A
Relative CARM1 level
20
15
10
5
0
**
***
****
PC9
A549
H1299
CARM1 in NSCLC cells
Enrichment of CARM1
Biontin-by miR-181a-5p
B
control
miR-181a-5p
Relative luciferase assay
2.0
1.5
1.0
0.5
0
**
ns
Wild type
Mutant
SIRT1
C
control
miR-181a-5p
Relative luciferase assay
1.5
1.0
0.5
0
*
ns
Wild type
Mutant
CDK8
D
control
miR-181a-5p
Relative luciferase assay
1.5
1.0
0.5
0
*
ns
Wild type
Mutant
IRS2
E
control
miR-181a-5p
Relative luciferase assay
2.0
1.5
1.0
0.5
0
**
ns
Wild type
Mutant
BHLHE40

Figure S5

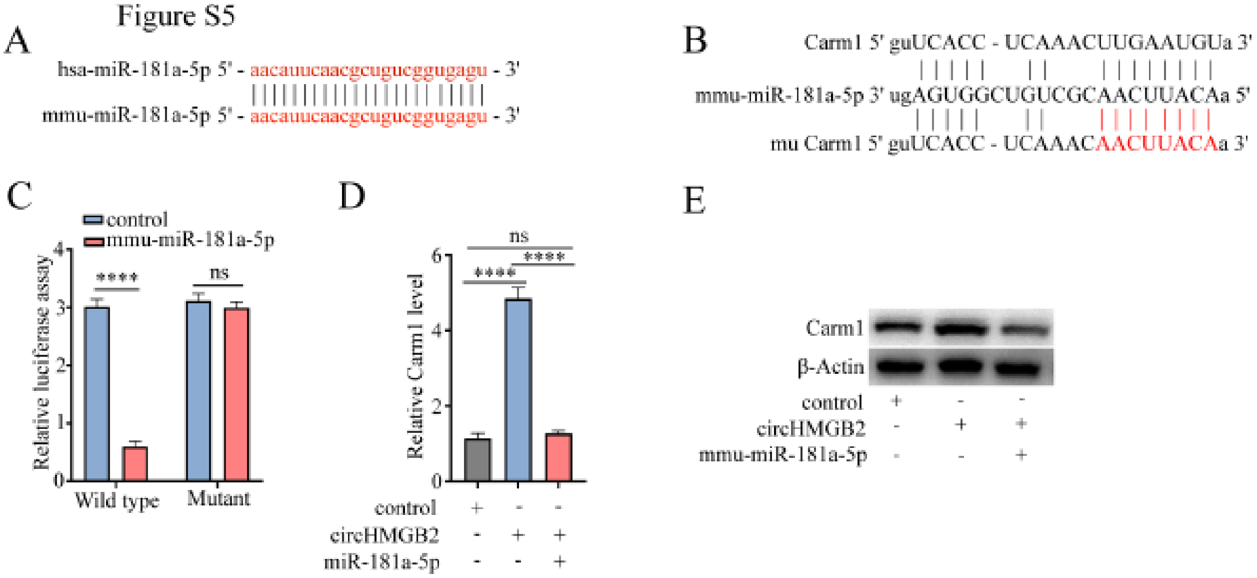

A
hsa-miR-181a-5p 5' - aacauucaacgcugucggugagu - 3'
mmu-miR-181a-5p 5' - aacauucaacgcugucggugagu - 3'
B
Carm1 5' guUCACC - UCAAACUUGAAUGUa 3'
mmu-miR-181a-5p 3' ugAGUGGCUGUCGCAACUUACAa 5'
mu Carm1 5' guUCACC - UCAAACAACUUACAa 3'
C
control
mmu-miR-181a-5p
Relative luciferase assay
****
ns
Wild type
Mutant
D
Relative Carm1 level
ns
****
****
control
circHMGB2
miR-181a-5p
E
Carm1
β-Actin
control
circHMGB2
mmu-miR-181a-5p

Figure S6

A

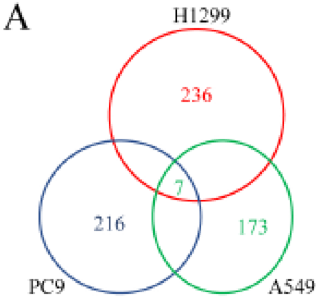

Accession	Description
Q86X55	Coactivator-associated arginine methyltransferase 1, CARM1
O15379	Histone deacetylase 3, HDAC3
P35579	Myosin-9, MYH9
P06899	Histone H2B type 1, J H2B1J
O00571	ATP-dependent RNA helicase DDX3X, DDX3X
P07355	Annexin A2, ANXA2
P17987	T-complex protein 1 subunit alpha, TCPA

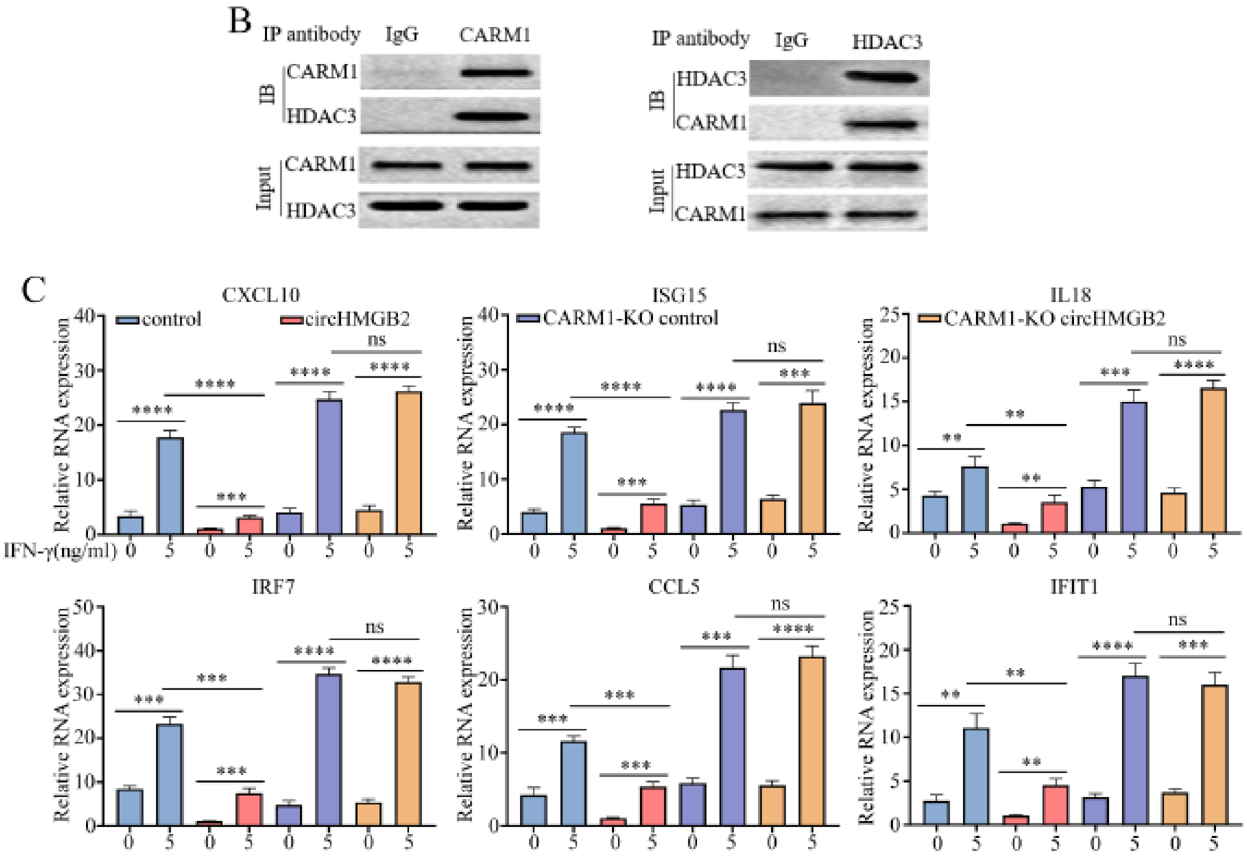

Supplement: Supplementary file 2 — Additional file 2: Supplementary Fig. 1. A. The expression of circUSP7 was measured in 3 pairs of NSCLC tissues and matched normal tissues. CircUSP7 was used as a positive control in this study. B. The expression of circHMGB2 was analyzed according to tumor diameter (< 2 cm vs. ≥ 2 cm), lymph node metastasis status (yes vs. no), and TNM stage in 78 LUAD patients. C, Survival analysis of the recurrence and OS of 78 LUAD patients divided into groups according to circHMGB2 expression (circHMGB2high vs. circHMBG2low) was performed using Kaplan–Meier and log rank analysis. Data are presented as the means ± SD; n = 3, *P < 0.05, **P < 0.01, ***P < 0.0001. Supplementary Fig. 2. A. The expression of circHMGB2 was measured in HBE, LLC and 5 NSCLC cell lines (NCI-H460, A549, PC9, H1703, and NCI-H1299 cells) using qRT–PCR. B. The transfection efficiency of three stable cell lines, A549-circHMGB2, H1299-shcircHMGB2 and LLC-circHMGB2, was validated by qRT–PCR. C. The invasion of A549-circHMGB2 and H1299-shcircHMGB2 cells was assessed by Matrigel Transwell assay. D. The migration of A549-circHMGB2 and H1299-shcircHMGB2 cells was assessed by wound healing assay. E, IHC staining of CD4+ T cells, Tregs, and TAM in subcutaneous tumors derived from LLC-control and LLC-circHMGB2 cells. Data are presented as the means ± SD; n = 3, ****P < 0.0001, ns: not significant. Supplementary Fig. 3. A. Electron microscopy image of exosomes in the supernatants of H460 cells. B. The levels of biomarkers of exosomes from the supernatants of NSCLC cells were measured by western blotting. C. The expression of circHMGB2 in exosomes derived from the supernatants of H460-control and H460-circHMGB2 cells was measured by qRT–PCR. Data are presented as the means ± SD; n = 3, ns: not significant. Supplementary Fig. 4. A. The RNA pulldown assay was performed with PC9, A549 and H1299 cells transfected with biotinylated miR-181a-5p. B, The luciferase activity of SIRT1 was measured in HEK-293 T cells transfected [file 12943_2022_1586_MOESM2_ESM.pdf]
